# A systematic analysis of the human immune response to *Plasmodium vivax*

**DOI:** 10.1172/JCI152463

**Published:** 2023-10-16

**Authors:** Florian A. Bach, Diana Muñoz Sandoval, Michalina Mazurczyk, Yrene Themistocleous, Thomas A. Rawlinson, Adam C. Harding, Alison Kemp, Sarah E. Silk, Jordan R. Barrett, Nick J. Edwards, Alasdair Ivens, Julian C. Rayner, Angela M. Minassian, Giorgio Napolitani, Simon J. Draper, Philip J. Spence

**Affiliations:** 1 Institute of Immunology and Infection Research, University of Edinburgh, Edinburgh, United Kingdom.; 2 Insitute of Microbiology, Universidad San Francisco de Quito, Quito, Ecuador.; 3 MRC Human Immunology Unit, Weatherall Institute of Molecular Medicine, and; 4 The Jenner Institute, University of Oxford, Oxford, United Kingdom.; 5 Cambridge Institute for Medical Research, University of Cambridge, Cambridge, United Kingdom.; 6 Department of Biochemistry, University of Oxford, Oxford, United Kingdom.

**Keywords:** Immunology, Infectious disease, Malaria

## Abstract

**BACKGROUND:**

The biology of *Plasmodium vivax* is markedly different from that of *P*. *falciparum*; how this shapes the immune response to infection remains unclear. To address this shortfall, we inoculated human volunteers with a clonal field isolate of *P. vivax* and tracked their response through infection and convalescence.

**METHODS:**

Participants were injected intravenously with blood-stage parasites and infection dynamics were tracked in real time by quantitative PCR. Whole blood samples were used for high dimensional protein analysis, RNA sequencing, and cytometry by time of flight, and temporal changes in the host response to *P. vivax* were quantified by linear regression. Comparative analyses with *P. falciparum* were then undertaken using analogous data sets derived from prior controlled human malaria infection studies.

**RESULTS:**

*P. vivax* rapidly induced a type I inflammatory response that coincided with hallmark features of clinical malaria. This acute-phase response shared remarkable overlap with that induced by *P. falciparum* but was significantly elevated (at RNA and protein levels), leading to an increased incidence of pyrexia. In contrast, T cell activation and terminal differentiation were significantly increased in volunteers infected with *P. falciparum*. Heterogeneous CD4^+^ T cells were found to dominate this adaptive response and phenotypic analysis revealed unexpected features normally associated with cytotoxicity and autoinflammatory disease.

**CONCLUSION:**

*P. vivax* triggers increased systemic interferon signaling (cf *P. falciparum*), which likely explains its reduced pyrogenic threshold. In contrast, *P. falciparum* drives T cell activation far in excess of *P. vivax*, which may partially explain why falciparum malaria more frequently causes severe disease.

**TRIAL REGISTRATION:**

ClinicalTrials.gov NCT03797989.

**FUNDING:**

The European Union’s Horizon 2020 Research and Innovation programme, the Wellcome Trust, and the Royal Society.

## Introduction

*Plasmodium vivax* causes more than half of all malaria cases in the Americas and Southeast Asia (1) and its distinct biology (cf *P. falciparum*) presents unique challenges for control and elimination. Dormant liver-stage parasites can trigger multiple relapses over many months or years (2); asexual blood-stage parasites preferentially accumulate in the spleen to promote chronic infection (3); and gametocytes develop rapidly within the bone marrow to maximize transmission (4). Total pathogen load is nevertheless reduced compared with *P. falciparum* and severe disease is a much less common outcome of infection (5). This may be explained in part by *P. vivax* invading CD71^+^ reticulocytes (as opposed to mature red cells) and sequestering far less effectively than *P. falciparum* (6, 7). But parasite virulence may also be influenced by the host response to infection (8). It is well established that the pyrogenic threshold is far lower for *P. vivax* (indicating differential regulation of the acute-phase response) and that clinical immunity (leading to asymptomatic infection) is acquired much more quickly (9–13). Parasite species may therefore regulate the immune response to shape the discrete patterns of disease observed in human malaria.

There are large gaps in our understanding of the immune response to *P. vivax* compared with the better-studied *P. falciparum* (14). We know the *P. vivax* genome is enriched in CpG motifs that can bind TLR9 to trigger type I interferon (IFN) production (15, 16) and that monocytes and neutrophils are highly phagocytic (and generate reactive oxygen species) during acute infection (17, 18). We also know that by invading reticulocytes (which express class I MHC), *P. vivax* may offer an alternative route to pathogen control — cytolysis by antigen-specific CD8^+^ T cells (19). Nevertheless, a systematic analysis of the immune response to *P. vivax* is lacking; we have limited information on the number or function of activated T cells in vivo, and field studies that directly compare the host response between *P. vivax* and *P. falciparum* are few and far between. These studies are extremely challenging in an endemic setting due to differences in host age, pathogen load, parasite genotype, and history of exposure. However, the resurgence of human challenge models (20–22) provides a unique opportunity to compare the immune response to these evolutionarily divergent parasites (23) while accounting for these important confounders.

Controlled human malaria infection (CHMI) has thus far shown that *P. vivax* triggers a systemic IFN-stimulated response (24) and activates the kynurenine pathway (likely through induction of IDO) (25). To build upon these findings, we generated a new cryopreserved stabilate of *P. vivax* suitable for CHMI (26) and used systems biology tools to map the immune response at unprecedented resolution. We then reasoned that a direct comparison with the host response to *P. falciparum* may shed new light on why falciparum malaria more frequently causes severe disease and why *P. vivax* is better able to induce rapid clinical immunity.

## Results

### P. vivax triggers IFN-stimulated inflammation.

**Figure 1:**
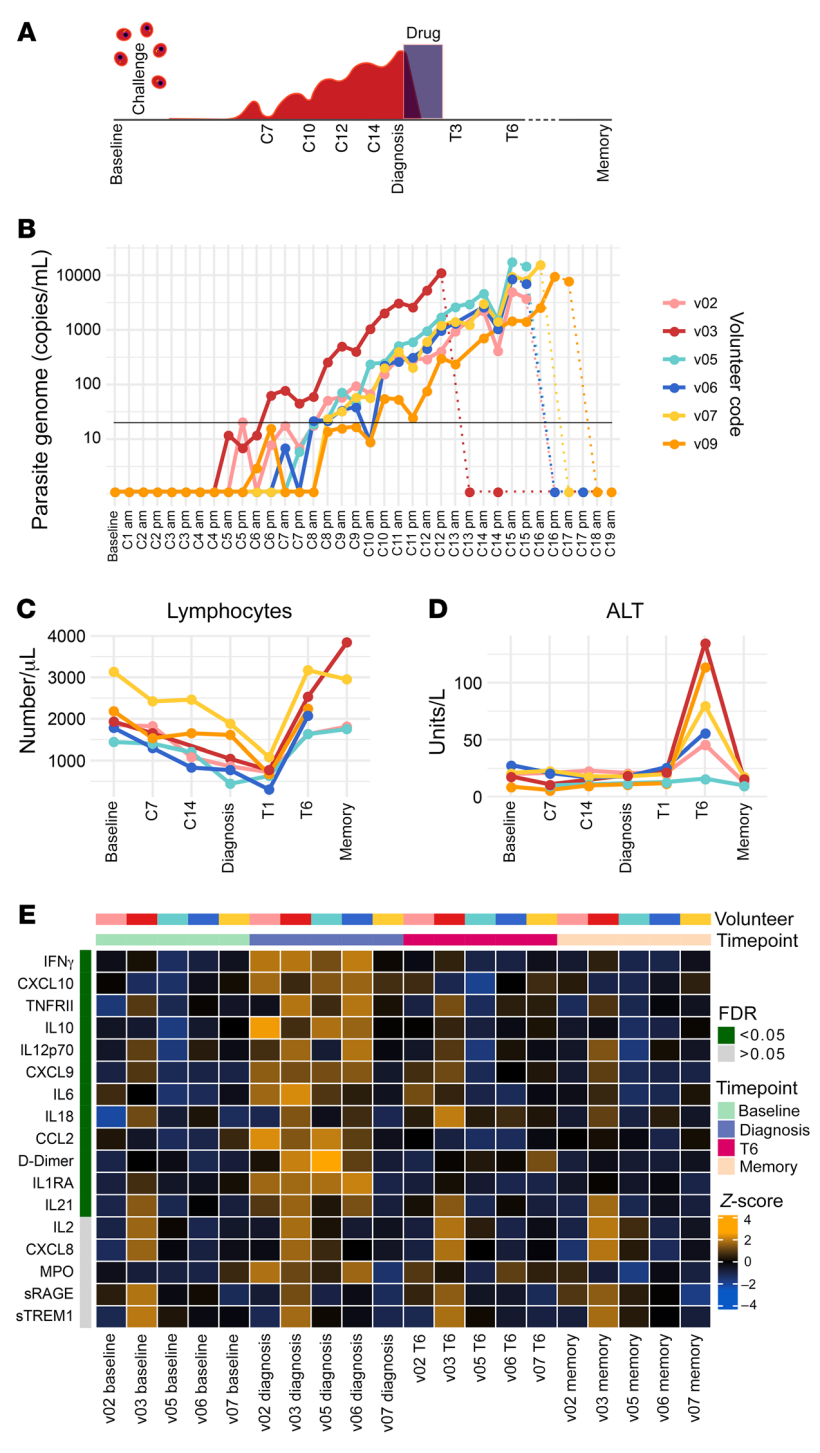
*Plasmodium vivax* triggers IFN-stimulated inflammation. (**A**) Study design and sampling time points. (**B**) Circulating parasite density was determined twice daily by qPCR. Pretreatment measurements are shown as solid lines, posttreatment measurements as dotted lines. The limit of quantification (20 genome copies/mL) is shown by a black line. (**C** and **D**) Full blood counts and blood chemistry measured (**C**) lymphocyte frequencies and (**D**) the concentration of alanine aminotransferase (ALT) throughout infection and convalescence. In **B**–**D**, each line represents 1 volunteer (*n* = 6). (**E**) Multiplexed plasma analytes were measured using a custom Legendplex assay. Each row in the heatmap is an analyte and each column a plasma sample. Samples from v09 were excluded after failing QC (*n* = 5). Linear regression was used to identify analytes that varied across the volunteer cohort at each time point (compared with baseline) and these are ordered by FDR. FDR < 0.05 was considered significant after adjusting for multiple testing (Benjamini-Hochberg). Only 17 of the 39 analytes measured are shown (those with the lowest FDR) and the color of each tile corresponds to the row-wise *z* score–transformed concentrations. In **C** and **D**, the memory time point is 90 days after challenge and in **E**, memory is 45 days after challenge.

Six malaria-naive volunteers were infected with *P. vivax* (clone PvW1) by direct blood challenge (Supplemental Table 1; supplemental material available online with this article; https://doi.org/10.1172/JCI152463DS1). These cryopreserved parasites originate from a naturally infected donor in Thailand and have been reset by mosquito transmission (see ref. 26 for details). All volunteers reached the treatment threshold (5,000 or 10,000 parasite genome copies per mL with or without symptoms, respectively) within 12–16 days of inoculation (Figure 1, A and B). Whole blood samples were taken at baseline (day before challenge), during infection (C7 for 7 days after challenge), at the peak of infection (diagnosis), after drug treatment (T6 for 6 days after treatment), and 45 days after challenge (memory time point). The majority of adverse events (including symptomatology, hematology, and blood chemistry) peaked within 24 hours of treatment. Notably, all volunteers exhibited pronounced lymphopenia (Figure 1C), serum transaminases were elevated in 5 of 6 volunteers, indicating liver injury (Figure 1D), and pyrexia was a common outcome of infection (26).

Next, we sought to capture the acute-phase response to *P. vivax* by quantifying 39 plasma analytes using a custom bead-based protein assay. To identify analytes that varied significantly through time, we fit linear regression models for each analyte in the form of analyte~timepoint+volunteer using log_10_-transformed concentrations and time point as a categorical variable. After correcting for multiple testing, we found 12 analytes with an FDR of less than 0.05 (Figure 1E). All significantly varying analytes increased in abundance and peaked at diagnosis (except IL-18, which peaked at T6). The analyte with the lowest FDR was IFN-γ, indicating a robust type I inflammatory response, which has been extensively described in febrile disease (including vivax malaria; refs. 24, 25, 27). In agreement, analytes associated with the recruitment (CCL2, CXCL9, and CXCL10) and activation (IL-12, IL-18, and IL-21) of inflammatory monocytes and CD4^+^ T cells were also induced. And furthermore, D-dimer (a biomarker of intravascular fibrinolysis that is intimately linked to systemic inflammation; ref. 28) was significantly increased. All analytes had returned to baseline levels by 45 days after challenge. Collectively, these data demonstrate that *P. vivax* triggers a type I inflammatory response that coincides with hallmark features of clinical malaria, including lymphopenia, pyrexia, and fibrinolysis. Importantly, symptoms and plasma analytes rapidly return to baseline levels after parasite clearance, with the exception of IL-18 and alanine aminotransferase (ALT), which peak 6 days after drug treatment.

### Inflammation is followed by proliferation in peripheral blood.

**Figure 2:**
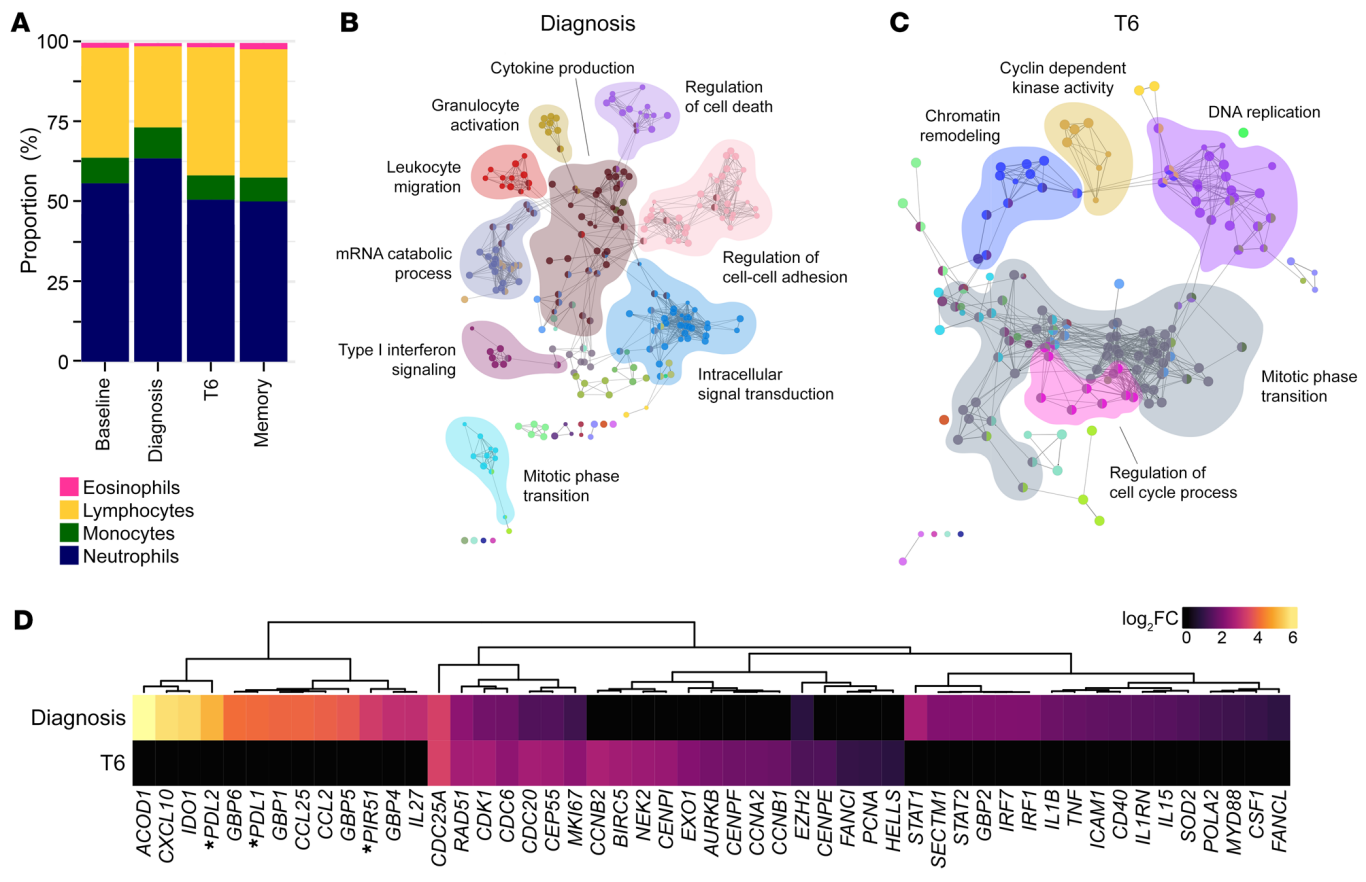
Inflammation is followed by a transcriptional signature of proliferation. (**A**) Proportion of lymphocytes, monocytes, neutrophils, and eosinophils in whole blood at baseline, diagnosis, T6, and 90 days after challenge (memory). The mean frequency is shown for each time point (*n* = 6). Note that the relative increase in abundance of myeloid cells between baseline and diagnosis is 13.6%. (**B** and **C**) Genes that were differentially expressed in whole blood at diagnosis (**B**) and T6 (**C**) (relative to baseline, *P*_adj_ < 0.05 and fold-change > 1.5) were used to create a gene ontology (GO) network in ClueGO. Each node represents a GO term and node size is determined by enrichment-adjusted *P* value. GO terms that share more than 40% of genes are connected by a line and organized into discrete functional groups (each given a unique color). The major functional groups are highlighted and labeled with a representative GO term. (**D**) The log_2_(fold-change) (log_2_FC) of signature genes associated with IFN signaling, type I inflammation, and proliferation are shown in whole blood at diagnosis and T6 (relative to baseline). Genes are ordered by unsupervised hierarchical clustering (denoted by the dendrogram) and those that were not differentially expressed (*P*_adj_ > 0.05) are shown with a fold-change of zero. Asterisks indicate that common gene names have been used. In **B**–**D**, *n* = 6 per time point.

To further characterize the systemic response to *P. vivax*, we used bulk RNA sequencing to resolve changes in whole blood gene expression through time. We first grouped samples by time point and then used DESeq2 (29) to perform pairwise comparisons with baseline samples. In this way, we could identify changes in gene expression that were shared across the volunteer cohort, and we used an adjusted *P* (*P*_adj_) value of less than 0.05 for significance. To take into account the drop in lymphocytes at diagnosis, we performed differential blood counts, which revealed that myeloid cells increased by 13.6% at the peak of infection (Figure 2A). We therefore only considered significant genes with an absolute fold-change greater than 1.5 so that differential expression could not merely be explained by lymphopenia (assuming myeloid/lymphoid cells share similar transcriptional activity). Using these 2 thresholds, we found that the transcriptional response peaked at diagnosis, with 2,221 differentially expressed genes (DEGs). Of note, there were no DEGs prior to diagnosis, indicating that *P. vivax* does not induce detectable changes in gene expression when infection is subpatent (below 5,000 to 10,000 parasites per mL). This is in line with previous observations (24). The number of DEGs dropped to 298 at T6 and surprisingly most of these were unique to this time point, suggesting that a distinct transcriptional response follows drug treatment.

To directly compare the biological functions of the host response at diagnosis and T6, we used gene ontology (GO) network analysis. ClueGO assigns significant GO terms based on differential gene expression and then groups them into functional networks by relatedness (30, 31). The transcriptional response at diagnosis was dominated by upregulation of innate signaling and defense pathways, including GO terms associated with NF-κB signaling, leukocyte migration, and cytokine production (Figure 2B). On the other hand, the GO terms unique to T6 related to chromatin remodeling and cell cycle progression rather than inflammation (Figure 2C). When we looked at the signature genes associated with these diverging networks, we found that the top hits at diagnosis were downstream targets of IFN signaling and critical regulators of host metabolism and T cell activation (for example, IDO and PDL1) (Figure 2D). Importantly, many of these genes have been shown to be upregulated in purified monocytes and neutrophils isolated from *P. vivax*–infected patients (17, 18). In contrast, the top DEGs after drug treatment included regulators of nuclear division and proliferation. This gene signature is unlikely to derive from activated myeloid cells, which are terminally differentiated and do not proliferate in peripheral blood. Instead, these data suggest that we are capturing activated lymphocytes as they return to the circulation 6 days after parasite clearance.

### Proliferation coincides with the appearance of activated T cells.

**Figure 3:**
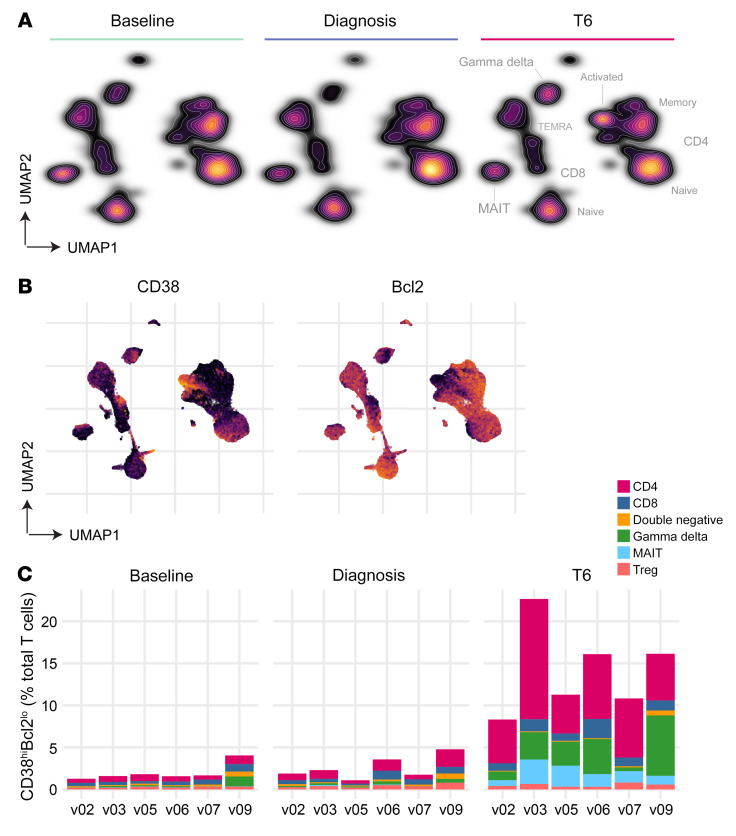
Proliferation coincides with the appearance of activated T cells. Whole blood was preserved within 30 minutes of blood draw at baseline, C10, diagnosis, and T6. Samples were stained with a T cell–focused antibody panel (details in Supplemental Table 2) and acquired on a Helios mass cytometer. (**A**) UMAP plot colored by cell density and split by time point; labels indicating the location of each major T cell subset are shown (refer to Supplemental Figure 1 for the expression of lineage and memory markers). (**B**) Expression of CD38 and Bcl2 across the UMAP projection at T6; each marker is independently scaled for visualization. In **A** and **B**, data from all volunteers were concatenated and split by time point (*n* = 6). (**C**) Stacked bar chart showing the sum of activated (CD38^hi^Bcl2^lo^) T cells at each time point; each bar represents 1 volunteer (*n* = 6) and bars are color-coded by lineage.

It is well known that T cells are recruited out of circulation during infection and activated within the inflamed spleen (32, 33); by studying their phenotype as they reenter peripheral blood, it may be possible to examine T cell priming and (by extension) the tissue environment in human malaria. To explore this idea, we leveraged cytometry by time of flight (CyTOF) to achieve single-cell resolution of T cell activation and differentiation. To examine these data, we first used uniform manifold approximation and projection (UMAP) (34) to visualize the phenotypic diversity of T cells at each time point. Cells close to each other in the UMAP space are phenotypically similar, whereas dissimilar cells are far apart. Remarkably, we found that the global structure of the T cell compartment appeared stable between baseline and diagnosis despite the profound loss of lymphocytes from peripheral blood (Figure 3A). On the other hand, a dense population of T cells appeared de novo at T6 when lymphocyte counts returned to baseline values (Figure 1C). Inspection of marker expression showed that these were predominantly CD4^+^ T cells with an effector memory (CD45RO^+^CCR7^–^) and activated (CD38^hi^Bcl2^lo^) phenotype (Figure 3B and Supplemental Figure 1). The latter marker combination revealed that *P. vivax* could activate up to one-quarter of the entire T cell compartment (Figure 3C).

**Figure 4:**
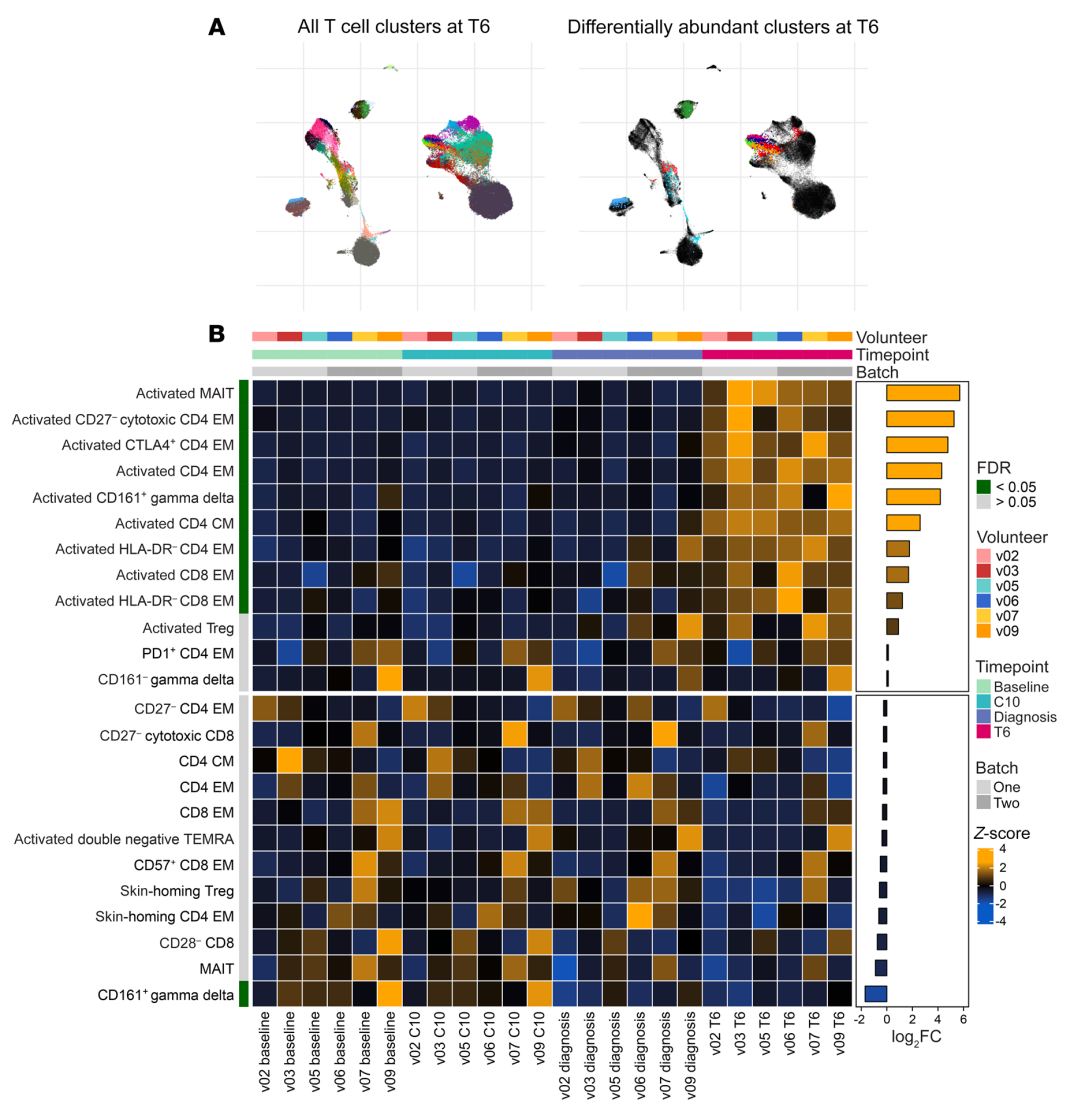
*Plasmodiumvivax* activates every T cell lineage. (**A**) UMAP plot showing all 34 T cell clusters (left) and those that were differentially abundant at T6 (right) (relative to baseline, FDR < 0.05 and fold-change > 2). Data from all volunteers and time points were concatenated for clustering, and each cluster has a unique color. (**B**) Heatmap showing the relative abundance of T cell clusters through time. Each row is a T cell cluster and each column a sample; clusters are ordered by log_2_(fold-change) (log_2_FC) at T6 (relative to baseline). Only 24 of the 34 T cell clusters are shown (those with the lowest FDR) and tiles are colored according to row-wise *z* scores of (arcsine square root–transformed) cluster frequencies. In **A** and **B**, *n* = 6 per time point. EM, effector memory; CM, central memory.

To comprehensively describe these phenotypic changes, we used FlowSOM clustering (35) to assign each T cell to one of 34 unique clusters and then tracked the frequency of each cluster through time (Supplemental Figures 2 and 3). To identify differentially abundant clusters at each time point (relative to baseline), we performed linear regression on cell count data using edgeR (36, 37). We found that no clusters were differentially abundant at C10 (FDR < 0.05 and absolute fold-change > 2) and only 1 cluster at diagnosis (a decrease in CD161^+^ γδ T cells). That only 1 of 34 clusters significantly changed in their relative size as the host became lymphopenic indicates that T cells are proportionally pulled out of circulation regardless of lineage or function. Using the same significance cutoffs, we identified 9 clusters that increased in abundance at T6, comprising 5 CD4^+^ and 2 CD8^+^ T cell subsets plus 1 mucosal-associated invariant T (MAIT) cell and 1 γδ T cell subset (Figure 4, A and B). Crucially, all displayed a CD38^hi^Bcl2^lo^ phenotype (Figure 3B and Figure 4A).

We then used a complementary method of analysis to examine differential marker expression through time, which can identify early activation events and phenotypic changes in cell subsets that may not increase in abundance (Supplemental Figure 4). This analysis revealed only very minor changes in circulating T cells at the peak of infection — a small increase in expression of CD38 on naive CD4^+^ T cells and the upregulation of T-bet in CD8^+^ terminally differentiated effector memory cells re-expressing CD45RA (Temra) and γδ T cells. In contrast, there were major phenotypic changes at T6, with the majority of differentially expressed markers (including death receptors and cytotoxic molecules) found on CD4^+^ and CD8^+^ T cells with a memory phenotype. Surprisingly, only 2 markers (Foxp3 and CTLA4) were differentially expressed on Tregs at T6 and none at diagnosis, which emphasizes the absence of overt regulatory pathways operating in peripheral blood. Altogether, these results suggest that innate-like and adaptive T cells are indiscriminately recruited out of the circulation and activated by *P. vivax*. The breadth and scale of T cell activation considerably exceeds what has been observed in other human challenge models, including typhoidal *Salmonella* (38) and influenza A (39).

### Activated T cells are functionally heterogeneous.

**Figure 5:**
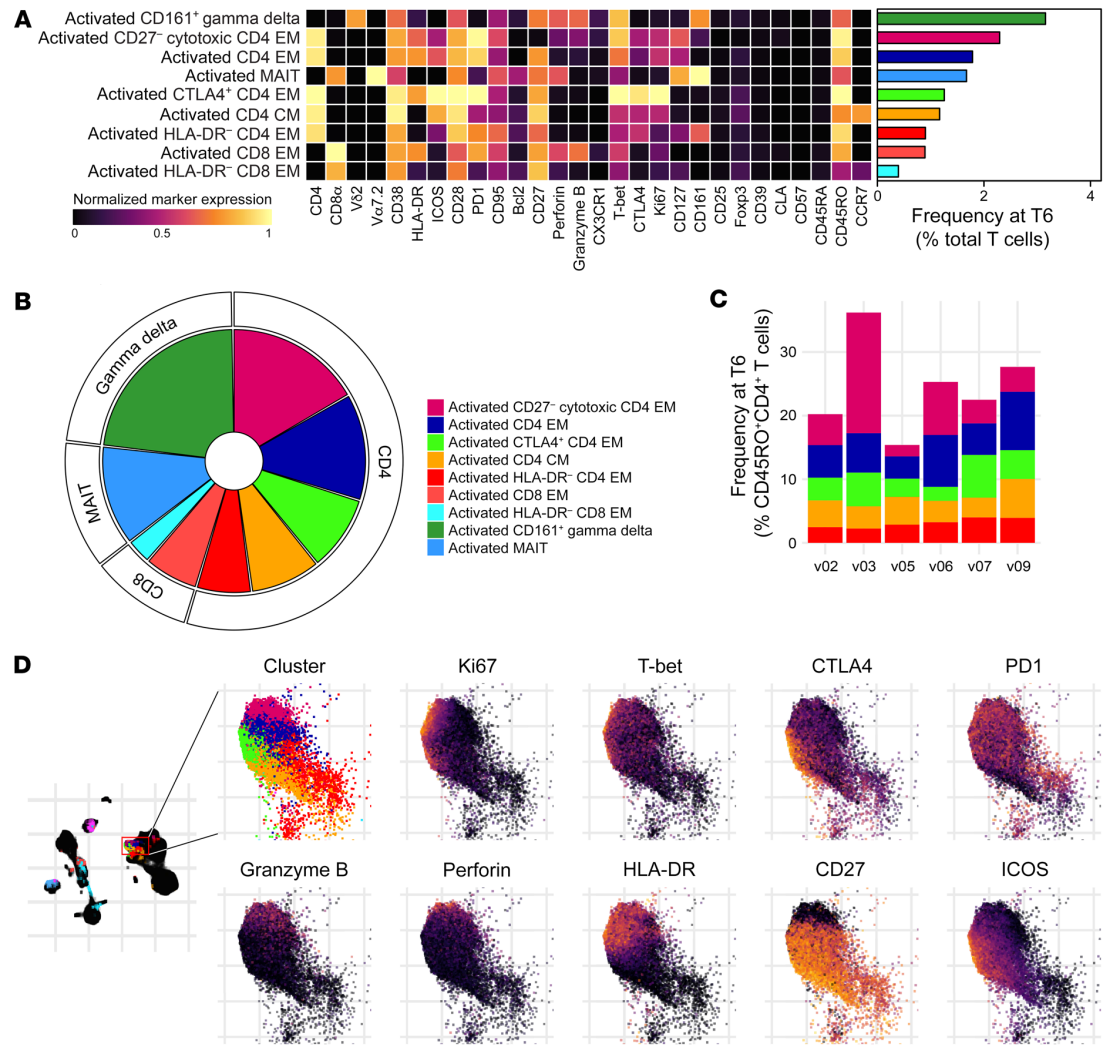
Activated T cells are functionally heterogeneous. (**A**) Heatmap showing normalized median expression values of all markers used for clustering in each of the 9 T cell clusters that were differentially abundant at T6. The horizontal bar chart shows the average frequency of each cluster across all volunteers. (**B**) Pie chart showing the relative size of each differentially abundant T cell cluster at T6. (**C**) Stacked bar chart showing the sum of activated CD4^+^ T cells at T6; each bar represents 1 volunteer. Data are shown as a proportion of the total non-naive CD45RO^+^CD4^+^ T cell pool. (**D**) UMAP plot showing the expression of activation, proliferation, and differentiation markers across each of the CD4^+^ T cell clusters that were differentially abundant at T6; each marker is independently scaled using arcsine-transformed signal intensity. The expression of these markers is shown across the entire UMAP plot in Supplemental Figure 5. In **A**–**D**, *n* = 6 and T cell clusters are color-coded according to the legend in **B**. EM, effector memory; CM, central memory.

To elucidate the function of activated T cells as they reenter the circulation, we inspected the median expression values of proliferation and differentiation markers in the 9 clusters that increased in abundance at T6 (Figure 5A and Supplemental Figure 5). And because we found that more than half of all activated T cells were CD4^+^ (with 5 distinct clusters contained within this lineage; Figure 5B), we focused on the heterogeneity of this adaptive response. High CD38 and low Bcl2 expression were shared features of all significant CD4^+^ clusters and 4 out of 5 displayed an effector memory phenotype (CD45RO^+^CCR7^–^). The one exception was a small cluster of activated central memory–like cells (CD45RO^+^CCR7^+^). By summing these 5 CD45RO^+^ clusters, we found that *P. vivax* activated 20%–35% of non-naive CD4^+^ T cells and the largest cluster had a CD27^–^ cytotoxic phenotype (perforin^+^granzyme B^+^) (Figure 5, A–C).

HLA-DR and ICOS were frequently upregulated on activated CD4^+^ T cells and we found widespread expression of inhibitory receptors, such as PD1 and CTLA4 (Figure 5D). These checkpoint inhibitors have been used as shorthand for exhaustion, and yet the majority of activated clusters were CD28^hi^, T-bet^+^, and proliferative (Ki-67^+^). Our data therefore suggested that these cells were functional and polarized toward an inflammatory T helper 1 (Th1) fate (Figure 5A). To specifically test for exhaustion or anergy at T6, we stimulated whole blood with PMA/ionomycin and quantified cytokine production ex vivo (Supplemental Figure 6). Activated CD38^hi^ T cells were polyfunctional and retained their capacity to produce all of the hallmark cytokines associated with Th1, Th2, Th17, and T follicular helper (Tfh) differentiation. *P. vivax* does not therefore exhaust activated CD4^+^ T cells, which can respond to mitogenic stimulation at least as well as resting (CD38^lo^) cells. In summary, CD4^+^ T cells with an effector memory phenotype dominate the response to *P. vivax* and display marked heterogeneity in their expression of key functional markers. These data therefore emphasize the complexity of CD4^+^ T cell activation and differentiation in vivax malaria.

### T cell activation is independent of systemic inflammation.

**Figure 6:**
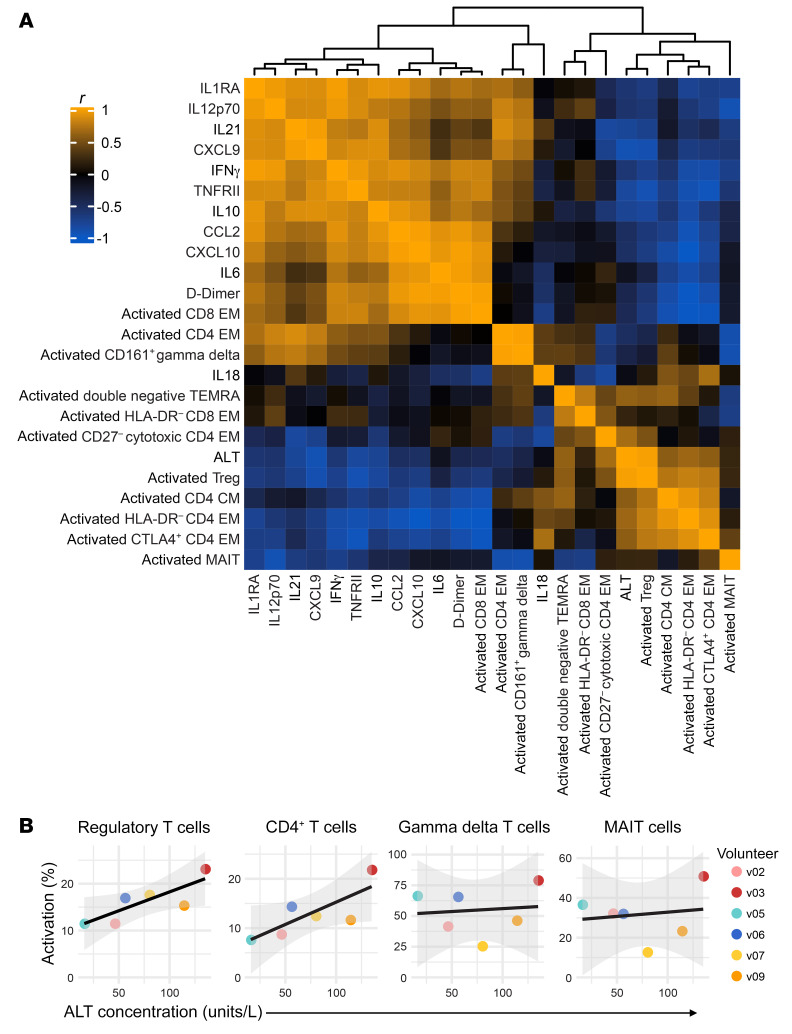
T cell activation is independent of systemic inflammation. (**A**) Heatmap showing a Pearson’s correlation matrix of the log_2_-transformed fold-change of each activated T cell cluster and the 12 most variable plasma analytes (FDR < 0.05). The fold-change was calculated either at diagnosis or T6 (relative to baseline) according to when this was largest for each feature. The absolute concentration of plasma ALT at T6 (the peak of the response) is also included. The order of features was determined by hierarchical clustering and the associated dendrogram is shown at the top of the heatmap. (**B**) Correlation between ALT concentration and the frequency of activated (CD38^hi^Bcl2^lo^) T cells at T6. Note that innate-like and adaptive T cell clusters belonging to the same lineage were merged to analyze their relationship with collateral tissue damage at a subset level. Regression lines are shown in black and the 95% confidence intervals in gray. In **A** and **B**, *n* = 6 per time point. EM, effector memory; CM, central memory.

Innate-like and adaptive T cells have distinct ligand requirements for T cell receptor (TCR) signaling, and yet every major T cell lineage was activated by *P. vivax* (Figure 4 and Supplemental Figure 4). We therefore hypothesized that the scale and breadth of the T cell response may indicate bystander (antigen-independent) activation, which can be caused by systemic inflammation (40, 41). To investigate the relationship between IFN-stimulated inflammation and T cell activation, we constructed a Pearson correlation matrix (Figure 6A). We input the log_2_(fold-change) of each plasma analyte with an FDR of less than 0.05 and the log_2_(fold-change) of each activated T cell cluster (defined as CD38^hi^Bcl2^lo^). Fold-change was calculated for each feature at either diagnosis or T6 (relative to baseline), depending on when the peak response was observed. Hierarchical clustering revealed extensive positive correlation between inflammatory cytokines, chemokines, and coagulation. In contrast, only 1 T cell cluster correlated highly with these analytes (*r* > 0.8 for activated CD8^+^ effector memory) and just 2 clusters showed weak correlations (activated CD161^+^ γδ and activated CD4^+^ effector memory). Instead, the majority of T cell clusters (8 of 11) were placed into a separate clade together with ALT, which indicates that the majority of the T cell response is coregulated but operates independently of systemic inflammation.

We next looked in detail at the relationship between T cell activation and ALT. Elevations in circulating ALT were positively correlated with the expansion of 4 activated CD4^+^ T cell clusters (including cytotoxic effector memory cells) as well as activated Tregs (*r* = 0.97). Because this analysis was looking for independent relationships with each cluster, we decided to repeat this analysis at a subset level. To this end, we calculated the correlation between lineage-specific T cell activation and absolute levels of ALT (Figure 6B). We found that ALT was strongly associated with activated CD4^+^ T cells (*r* = 0.791) and Tregs (*r* = 0.816) but not innate-like MAIT (*r* = 0.147) or γδ T cells (*r* = 0.107). These data therefore reveal no clear relationship between the intensity of systemic inflammation at diagnosis and the magnitude of the T cell response at T6. Instead, they indicate that CD4^+^ T cell activation may accurately predict collateral tissue damage and injury.

### Parasite species regulate T cell activation and differentiation.

**Figure 7:**
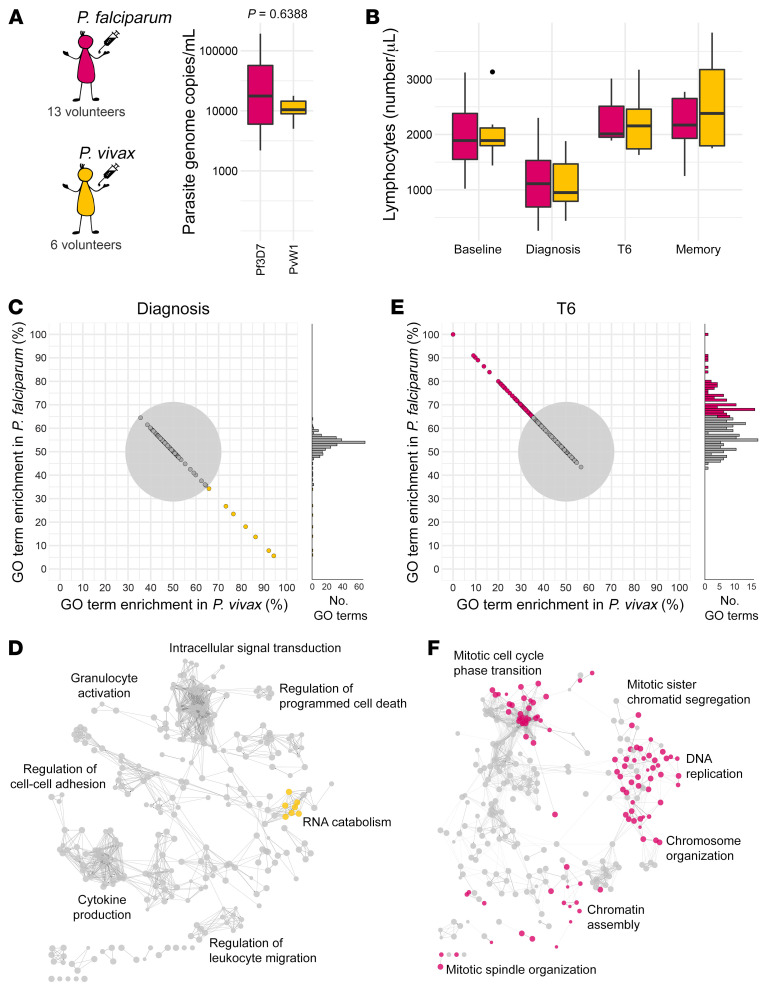
The host response is shaped by parasite species. (**A**) The maximum circulating parasite density in each volunteer during the VAC063/VAC063C CHMI trials (*Plasmodium falciparum*) and the VAC069A study (*Plasmodium vivax*). Significance between parasite species was assessed by 2-tailed Wilcoxon’s rank-sum exact test. (**B**) The total number of circulating lymphocytes through infection and convalescence; the memory time point is 90 days after challenge. In **A** and **B**, box (median and IQR) and whisker (1.5× upper or lower IQR) plots are shown with outliers as dots; *n* = 13 for *P. falciparum* and *n* = 6 for *P. vivax* (except at T6, where *n* = 3 for *P. falciparum*). (**C**–**F**) Whole blood RNA sequencing was performed identically during the VAC063/VAC063C and VAC069A studies and lists of differentially expressed genes (*P*_adj_ < 0.05 and fold-change > 1.5) were combined for GO analysis at diagnosis and T6. Importantly, for every GO term the fraction of associated genes derived from each volunteer cohort was retained. (**C** and **E**) Each GO term is represented by a single point and these are positioned according to the proportion of genes that were differentially expressed in volunteers infected with *P. falciparum* or *P. vivax*. The gray circle represents a 65% threshold that needed to be crossed to call a GO term as predominantly derived from 1 volunteer cohort; beyond this threshold GO terms are colored by enrichment as shown in **A**. (**D** and **F**) ClueGO networks reveal the functional organization of GO terms at diagnosis (**D**) and T6 (**F**); nodes are color-coded by enrichment (shared GO terms are shown in gray) and each of the major functional groups is labeled with a representative GO term. In **C** and **D**, *n* = 13 for *P. falciparum* and *n* = 6 for *P. vivax* and in **E** and **F**, *n* = 3 and 6, respectively.

Last of all, we performed direct comparative analyses to ask whether the immune response to *P. vivax* differed from that to *P. falciparum*. Thirteen malaria-naive volunteers were infected with *P. falciparum* (clone 3D7) by direct blood challenge during the VAC063 (42) and VAC063C (43) CHMI trials; crucially, diagnosis and treatment thresholds were analogous to VAC069A (*P. vivax*) and circulating parasite densities were comparable at the peak of infection (Figure 7A and Supplemental Figure 7). Moreover, the magnitude and kinetics of lymphopenia were equivalent between parasite species (Figure 7B). Initially, we compared transcriptional signatures in whole blood using time-matched samples. DEGs were identified at diagnosis and T6 (relative to baseline) in both volunteer cohorts using DESeq2 (*P*_adj_ < 0.05 and absolute fold-change > 1.5). The DEGs from each cohort were then combined at each time point to identify significantly enriched GO terms and functional network analysis was performed using ClueGO. Importantly, information was retained to indicate what fraction of associated genes for each GO term derived from *P. vivax*– or *P. falciparum*–infected volunteers.

At diagnosis we found 289 GO terms, of which 282 (97.58%) were shared between cohorts (Figure 7C). These shared GO terms organized into functional groups that related to host defense and cytokine production (Figure 7D). Remarkably, we found only 7 GO terms (2.42%) with associated genes that were predominantly derived from 1 volunteer cohort. All of these cohort-specific GO terms were located in the same region of the ClueGO network and were enriched in volunteers infected with *P. vivax* — this response was characterized by downregulation of structural ribosomal gene expression, which can be induced by type I IFN signaling (44). In contrast to diagnosis, only 151 out of 235 (64.3%) GO terms were shared at T6 and these features related to cell cycle progression (Figure 7, E and F). All of the remaining GO terms (84/235 or 35.7%) were predominantly derived from just 1 data set (*P. falciparum*) and these terms were accessory to cell division, such as DNA replication. These data therefore suggest that *P. vivax* may trigger increased IFN signaling at the peak of infection, but *P. falciparum* drives a much stronger proliferative response, which is observed when lymphocytes return to the circulation after parasite clearance.

**Figure 8:**
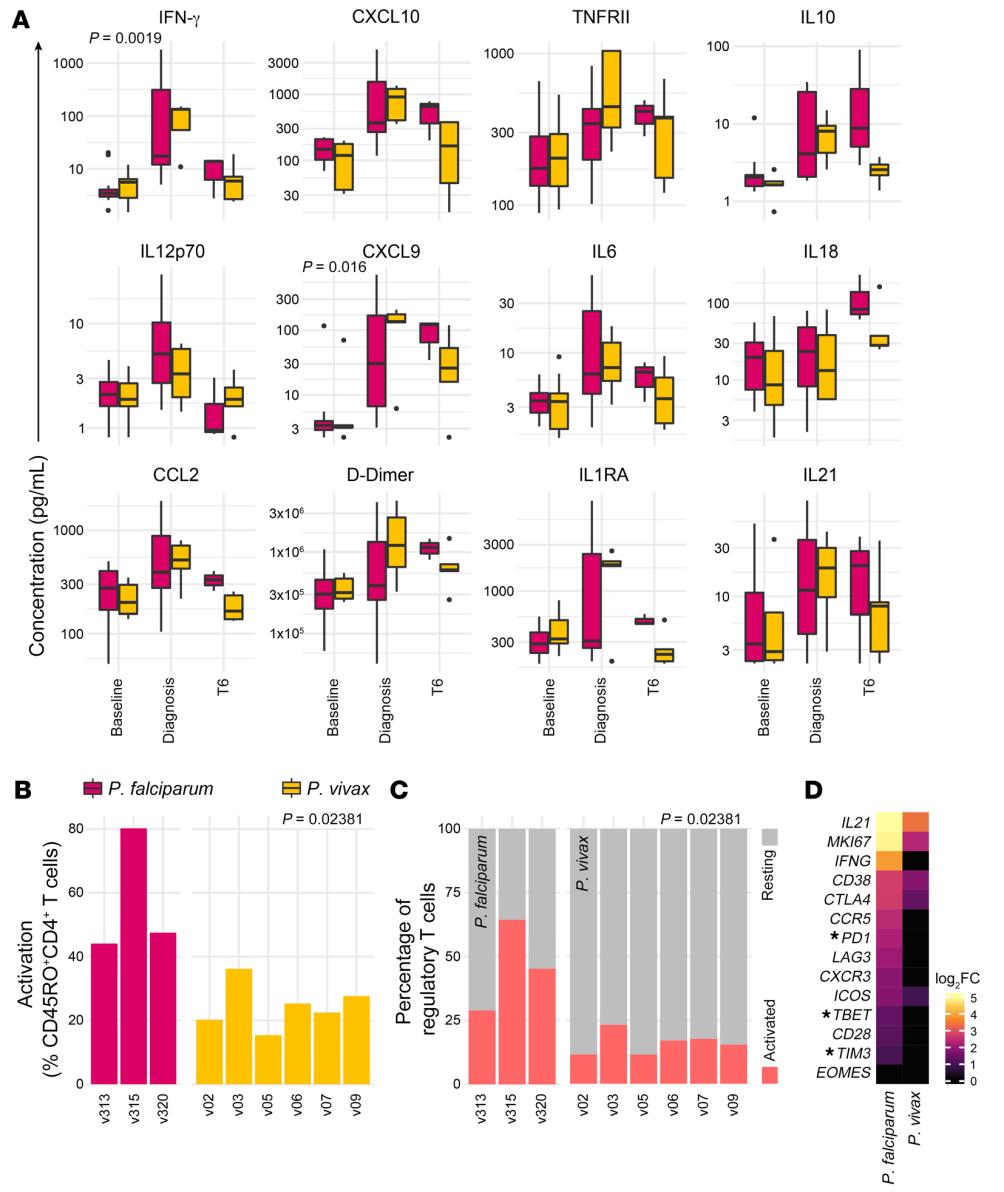
Parasite species regulate T cell activation and differentiation. (**A**) Multiplexed plasma analytes were measured in the VAC063/VAC063C and VAC069A CHMI studies using a custom Legendplex assay. Linear regression was used to identify analytes that vary significantly between volunteer cohorts at diagnosis and/or T6 (relative to baseline). After correcting for multiple comparisons (Benjamini-Hochberg), only 2 of 39 analytes were significant (*P*_adj_ < 0.05 at diagnosis). The 12 plasma analytes shown all varied significantly through time in *Plasmodium vivax*–infected volunteers (as shown in Figure 1E). Box (median and IQR) and whisker (1.5× upper or lower IQR) plots are shown with outliers as dots; *n* = 12 for *Plasmodium falciparum* at baseline/diagnosis and *n* = 3 at T6; *n* = 5 for *P*. *vivax* at all time points. Note that samples from v1040 (VAC063) and v09 (VAC069A) were excluded after failing QC. (**B** and **C**) The proportion of non-naive CD45RO^+^CD4^+^ T cells (**B**) and Tregs (**C**) activated (CD38^hi^Bcl2^lo^) at T6 in volunteers infected with *P. falciparum* or *P. vivax*. FlowSOM was used to identify activated T cell clusters independently in each volunteer cohort and the frequency of activated clusters were summed; each bar represents 1 volunteer. Significance between parasite species was assessed by 2-tailed Wilcoxon’s rank-sum exact test. (**D**) Heatmap of signature T cell genes showing their log_2_(fold-change) (log_2_FC) at T6 (relative to baseline) in whole blood analyzed by RNA sequencing; *n* = 3 for *P. falciparum* and *n* = 6 for *P. vivax*. Asterisks indicate that common gene names were used and genes that were not differentially expressed (*P*_adj_ > 0.05) are shown with a fold-change of zero.

To explore these possibilities, we compared the acute-phase response to *P. vivax* and *P. falciparum* using a bead-based protein assay and the CD4^+^ T cell response by CyTOF. First, we used mixed-effects models to quantify differences in systemic inflammation and coagulation at diagnosis and T6. We found a significant increase in IFN-γ and the IFN-responsive chemokine CXCL9 in volunteers infected with *P. vivax* (Figure 8A). Surprisingly, none of the 39 plasma analytes were significantly higher in volunteers infected with *P. falciparum* at either time point. In contrast, CyTOF revealed that *P. falciparum* drives increased activation of CD4^+^ T cells and Tregs (Figure 8, B and C). This adaptive response was so pronounced that a clear transcriptional signature of Th1 polarization could be detected in whole blood in volunteers infected with *P. falciparum* (but not *P. vivax*) (Figure 8D). Altogether, these data demonstrate that *P. vivax* can induce higher levels of systemic inflammation, but *P. falciparum* promotes increased CD4^+^ T cell activation and terminal differentiation.

## Discussion

High-dimensional protein and RNA sequencing data show that *P. vivax* can induce an enhanced IFN-stimulated response (cf *P. falciparum*) in a naive human host, which likely explains its reduced pyrogenic threshold. Indeed, we have recorded fever in 17 out of 19 (*P. vivax*) versus 23 out of 39 (*P. falciparum*) volunteers in all of our blood-stage CHMI studies (89.5% versus 59.0%, *P* = 0.0168 by Barnard’s unconditional exact test [includes unpublished trials]). This is unlikely to be explained by the increased time taken to reach the treatment threshold with *P. vivax* (Supplemental Figure 7) because there is no measurable transcriptional response prior to patency (as shown in this study and others; ref. 24). Instead, we suggest that this is a consequence of more sensitive pathogen sensing by innate immune sentinels. In small animal models, malaria parasites are first detected in the bone marrow by plasmacytoid dendritic cells, which produce type I IFN to trigger emergency myelopoiesis and the release of activated monocytes and neutrophils (45, 46). This model is well supported by transcriptional evidence of IFN signaling in the bone marrow of macaques infected with *P. cynomolgi* (47) and the accumulation of myeloid cells with an IFN-stimulated gene signature in the circulation of *P. vivax*–infected patients (17, 18). The transcriptional changes we observe at diagnosis are therefore likely to represent the trafficking of activated monocytes and neutrophils from bone marrow to blood. So why would this innate response differ between *P. vivax* and *P. falciparum*? Perhaps *P. vivax* accumulates in higher numbers within the bone marrow parenchyma (4), increasing the availability of parasites for uptake by immune sentinels. Together with the increased abundance of CpG motifs in the *P. vivax* genome (15, 16), this may enhance TLR9 signaling and in turn amplify the production of type I IFN. Sampling of bone marrow in future CHMI studies will allow us to directly test this hypothesis in vivo.

A key function of IFN-stimulated inflammation is the release of chemotactic factors that recruit T cells out of the circulation and into inflamed tissues, including the spleen. We found a significant increase in plasma CXCL9 and CXCL10 at the peak of infection, which coincided with profound lymphopenia. The surface-bound receptor for these chemokines is CXCR3, which is a hallmark of Th1-polarized memory CD4^+^ T cells and cytotoxic effector CD8^+^ T cells (48). As such, we expected that these T cell subsets would preferentially be recruited out of the circulation and were surprised to find that the relative abundance of every T cell cluster was essentially unchanged at diagnosis. Our data therefore reveal an indiscriminate mechanism of recruitment that pulls all T cells (regardless of lineage or function) out of peripheral blood. Furthermore, it was surprising that we found almost no evidence of T cell activation at diagnosis (either by cluster abundance or differential marker expression). Evidently, activated T cells do not commonly recirculate during infection and this has major implications for human studies that analyze adaptive responses prior to drug treatment. In the absence of direct access to tissue samples, T cell function should be assessed after their release back into the circulation, which occurs 6 days after parasite clearance. At this time point we find that up to one-quarter of the entire T cell compartment is activated, including half of all γδ T cells. How does infection trigger such widespread activation? This is unlikely to be a direct xenobiotic effect of antimalarial drugs, as artemisinin and its derivatives have been shown to inhibit T cell responses in a dose-dependent manner in vitro and in vivo (49, 50). Instead, an important clue might derive from the activation of MAIT cells, which recognize riboflavin-derived antigens presented on an MHC-like molecule (51). Malaria parasites can not synthesize riboflavin, which suggests that MAIT cells are not responding in an antigen-specific manner but acting as sensors of tissue inflammation (they can be activated directly by IL-12 and IL-18; ref. 41). Adaptive T cells can also be activated via this route (40), raising the intriguing possibility that *P. vivax* may cause bystander activation of human T cells. In support of this idea, TCRβ sequencing in mice infected with *P. chabaudi* has revealed polyclonal activation of CD4^+^ T cells within the inflamed spleen (52).

Activated CD4^+^ T cells account for more than half of the T cell response to *P. vivax* and all have a memory (CD45RO^+^) phenotype. This may indicate that CD4^+^ T cells specific for irrelevant pathogens (or vaccine epitopes) are activated and that we are observing the clonal expansion of preexisting memory cells. Compared with naive T cells, memory cells are more easily activated in the absence of TCR signals (40, 53). Alternatively, these may not be bona fide memory cells but instead short-lived effectors that are specific for a large and diverse pool of *Plasmodium* epitopes. Future studies should therefore prioritize investigating the antigen specificity and clonality of activated cells; this is particularly important because effector T cells and Tregs activated via TCR signals may be functionally distinct from those activated through bystander mechanisms. In the meantime, our data show that the dominant CD4^+^ T cell cluster displayed a terminally differentiated CD27^–^ cytotoxic phenotype; cells pushed down this route of differentiation typically arise in the context of chronic stimulation or autoinflammatory disease (54–56). These results therefore highlight the potency of activating signals within inflamed tissues and the potential pathogenicity of the T cell response to *P. vivax*.

Nevertheless, our data do not support a model of infection-induced exhaustion. Most of the CD38^hi^ T cell clusters upregulated inhibitory receptors in vivo and yet retained their capacity to respond vigorously to mitogenic stimulation and produce functionally diverse cytokines ex vivo. Similar observations have been made in *P. vivax*–infected patients (57). It remains possible that features of exhaustion may manifest during chronic infection, but at present there is little evidence to support T cell dysfunction in acute disease. Similarly, there is little evidence that Tregs suppress effector responses early in infection. Tregs obtained from *P. vivax*–infected patients have a reduced capacity to control CD4^+^ T cell proliferation and IFN-γ production in suppressor assays (58), and in our study the abundance of activated Tregs did not significantly increase at diagnosis or T6. Functional Tregs may operate within inflamed tissues (and this should be assessed after their return to the circulation), but it seems likely that regulatory networks are simply overwhelmed by the Th1-polarized effector response (as observed for *Toxoplasma gondii*; ref. 59).

So what are the likely consequences of this aggressive T cell response for the course and outcome of infection? The production of class-switched IgG antibodies specific for the merozoite protein MSP1_19_ indicates functional B cell help in our study (26). Unfortunately, we have not yet been able to examine the frequency or function of circulating Tfh cells at T6. Nonetheless, circulating CD4^+^ T cells with a Tfh phenotype have been reported in patients infected with *P. vivax* and this coincided with an increase in plasma IL-21 (60), which was significantly upregulated in our volunteers. Whether these antibodies can effectively neutralize infected reticulocytes in vivo could be assessed by reinfecting volunteers. The direct cytolysis of infected reticulocytes by activated cytotoxic CD8^+^ T cells also requires validation in vivo. What does seem clear, however, is that the adaptive T cell response correlates closely with collateral tissue damage. Liver injury is a common feature of clinical and experimental malaria and occurs independently of the drug used or treatment regime (61, 62). It has been suggested that systemic inflammation might trigger hepatocyte death, but our data do not support this hypothesis; we find no relationship between cytokine/chemokine levels and raised ALT. Instead, we propose that T cell trafficking to the liver (which is commonly observed during the resolution of an immune response and facilitates the clearance of apoptotic T cells; ref. 63) may lead to off-target cytotoxicity. In agreement, increased ALT in macaques infected with *P. cynomolgi* coincides with inflammatory infiltrates (including lymphocytes) in the liver (64).

Remarkably, CyTOF and RNA sequencing data show that *P. falciparum* causes increased activation and polarization of CD4^+^ T cells (cf *P. vivax*). How do we reconcile increased T cell activation with lower systemic inflammation? Parasitemia was comparable between volunteer cohorts, but it remains possible that the total pathogen load was higher for *P. falciparum* because of increased accumulation in inflamed tissues. An alternative explanation is that *P. falciparum* encodes more immunogenic proteins that can cross-react with preexisting memory T cells. In any case, more T cell activation may be one of the reasons why *P. falciparum* more frequently causes severe disease. As infection progresses, an excess of activated T cells could exacerbate inflammation, coagulation, and collateral damage to promote endothelium activation and sequestration in their tissue environment. In this context, it is important to note that the pathogenicity of T cells in infants and children has not yet been adequately assessed after their return to the circulation (65). Furthermore, the disconnect between systemic inflammation and tissue-specific responses might explain why field studies consistently fail to find reproducible associations between circulating protein markers of inflammation and disease severity.

Could increased T cell activation also explain why clinical immunity takes far longer to develop against *P. falciparum*? After all, this may lead to increased exhaustion if infection is not curtailed by drug treatment. Clinical immunity requires emergency myelopoiesis to be disarmed within the bone marrow to prevent the release of activated monocytes and neutrophils (and pyrogenic cytokines). Immune sentinels therefore need to tolerate malaria parasites (or become refractory to IFN signaling) and this adaptation may require T cell help (45). Alternatively, clinical immunity might be promoted by infection-induced remodeling of the bone marrow or reprogramming of innate immune progenitors (66). In this scenario, the accumulation of *P. vivax* in the bone marrow parenchyma might have an associated cost (a reduced pyrogenic threshold) but the benefit of faster clinical immunity. Developing human challenge models that incorporate reinfection and tissue sampling would allow us to ask how parasite biology shapes the discrete patterns of disease observed in human malaria.

### Limitations of the study.

We measure shared (or common) responses to *P. vivax* at a group level and could not quantify the heterogeneity between volunteers, which is likely to be a critical determinant of infection outcome. Indeed, our plasma data suggest that v07 does not trigger a detectable inflammatory response at diagnosis, which is in line with our observation of immune quiescence in one-third of volunteers undergoing CHMI with *P. falciparum* (67). On the other hand, we find that every volunteer triggers widespread activation of innate-like and adaptive T cells (including v07), and terminally differentiated CD27^–^ cytotoxic CD4^+^ T cells are a conserved feature across the cohort. Our results therefore emphasize the disconnect between systemic inflammation and T cell activation in human malaria. Nonetheless, future studies should increase sample size so that the host response can be mapped through time in each individual to identify unusual outcomes. Human T cells are inherently plastic (68) and a larger sample size is likely to reveal further diversity and identify rare T cell phenotypes that might be critical for pathogenesis. In the meantime, caution should be taken not to generalize these findings to every individual infected with *P. vivax*. A second important caveat is that infections were terminated at a parasite density much lower than would be observed in endemic settings. The impact of increasing pathogen load on T cell activation (or exhaustion) is difficult to predict and we are not aware of any posttreatment data from patients infected with *P. vivax* or *P. falciparum*. This is a critical knowledge gap that urgently needs to be addressed. Moreover, the pathogen load within lymphoid tissues (such as bone marrow or spleen) might be a more important determinant of the host response than the number of circulating parasites, but at present it is not possible to quantify this. Direct tissue access (via fine-needle aspiration; ref. 69) would overcome this limitation and reveal the biology of T cell activation where it matters most.

## Methods

Note that additional details regarding methodology and statistical analysis can be found in the Supplemental Methods.

### VAC069A clinical trial

Six volunteers were recruited to test the infectivity of a new cryopreserved stabilate containing a clonal field isolate of *P. vivax* (PvW1); this stabilate had carefully been prepared for use in CHMI by blood challenge. The CHMI trial was named VAC069A and is reported in full in Minassian et al. (26). In brief, cryopreserved vials of stabilate were thawed, washed, and diluted under aseptic conditions, and then administered to healthy malaria-naive adult volunteers by intravenous injection. Volunteers each received an inoculum containing between 116 and 2232 PvW1 genome copies; variation in the infectious dose had no measurable effect on parasite multiplication rate (26) and does not influence the severity or outcome of human malaria (70). After inoculation, whole blood was drawn twice daily to determine circulating parasite density by qPCR (target gene = 18S ribosomal RNA); thick blood smears were also evaluated by experienced microscopists at each time point. Treatment was initiated once 2 diagnostic conditions were fulfilled: more than 5,000 or 10,000 parasite genome copies/mL, positive thick blood smear, and/or symptoms consistent with malaria. Treatment usually consisted of artemether and lumefantrine (Riamet) or atovaquone and proguanil (Malarone) if Riamet was contraindicated. Volunteer 05 received Malarone; all other volunteers received Riamet.

All reported clinical symptoms (arthralgia, back pain, chills, diarrhea, fatigue, fever, headache, malaise, myalgia, nausea, pyrexia, rigor, sweating, and vomiting) were recorded as adverse events and assigned a severity score: 1, transient or mild discomfort (no medical intervention required); 2, mild to moderate limitation in activity (no or minimal medical intervention required); and 3, marked or severe limitation in activity requiring assistance (may require medical intervention). At baseline, C7, C14 (if undiagnosed), diagnosis, T1, and T6 full blood counts and blood chemistry were evaluated at the Churchill and John Radcliffe hospitals in Oxford, providing 5-part differential white cell counts and quantification of electrolytes, urea, creatinine, bilirubin, ALT, alkaline phosphatase (ALP), and albumin. Blood for immunological analyses was collected in EDTA tubes by venipuncture at the indicated time points, and samples were processed immediately for downstream applications in a laboratory adjacent to the clinical facility.

### CyTOF sample acquisition

Whole blood samples were taken at baseline, C10, diagnosis, and T6 and stabilized in whole blood preservation buffer (Cytodelics AB) within 30 minutes of blood draw. Preserved samples were stored at –80°C. Samples were thawed in a water bath at 37°C and then fixed and red cells lysed using the whole blood preservation kit (Cytodelics AB). Fixed samples were washed, permeabilized, and barcoded using the cell-ID 20-plex Pd barcoding kit (Fluidigm). Barcoded samples were then pooled and counted before resuspending in cell staining buffer (Fluidigm) at 40 × 10^6^ cells/mL. An equal volume of freshly prepared antibody cocktail (Supplemental Table 2) was added for 30 minutes at room temperature under gentle agitation. After washing, samples were resuspended in Nuclear Antigen Staining Buffer (Fluidigm) for 30 minutes at room temperature. Samples were then washed in Nuclear Antigen Staining Perm Buffer (Fluidigm) and antibodies for intracellular/nuclear targets were added for a further 45 minutes. After another round of washing, the cells were fixed with 1.6% paraformaldehyde in PBS for 10 minutes and finally resuspended in Fix and Perm Buffer plus 72.5 nM cell-ID 191Ir/193Ir intercalator (both Fluidigm) at a concentration of 2 × 10^6^ cells/mL. Cells were incubated overnight at 4°C. Sample acquisition was performed on a freshly tuned Helios mass cytometer using the WB injector and acquired with 10% normalization beads (140Ce, 151Eu, 165Ho, and 175Lu, all Fluidigm). Both staining and sample acquisition were carried out in 2 batches (all time points for 3 volunteers per batch). On each acquisition day, pooled cells were counted again before removing an aliquot of 2 × 10^6^ cells; aliquots were washed twice in cell staining buffer and resuspended in 1 mL Cell Acquisition Solution (Fluidigm). Each aliquot was acquired completely before washing and processing the next aliquot until all pooled samples had been acquired. Cells were acquired at a rate of 300–500 events per second.

### CyTOF data processing

FCS files were generated using CyTOF software (v6.7; https://www.standardbio.com/products/software) followed by normalization (71) and debarcoding (72) using the CATALYST workflow (github.com/HelenaLC/CATALYST) described in Nowicka et al. (73). Single-stained beads were used for compensation (using non-negative linear least squares regression; ref. 74) and FCS files were gated in the Cytobank web portal (Beckman Coulter) to exclude normalization beads and doublets. Singlet T cells (CD45^+^CD3^+^) were taken forward for analysis. Intensity distributions of each channel were inspected to remove channels of low variance (CD14, Tim3, integrin β7, CD56, CD16, CD49d, CD103, CXCR5). Of note, low variance in these channels does not necessarily reflect uniform or absent expression of these markers; it could also be due to inefficient staining of fixed samples with these antibody clones. The remaining 28 markers were used for both UMAP plots and FlowSOM clustering.

UMAP (34) creates low-dimensional projections of high-dimensional data. Here, cells were grouped according to marker expression intensity and embedded in a 2D plane such that phenotypic similarity within and between populations is preserved in the Euclidean distance of the projection. We used its R implementation in the scater package (75), which in turn relies on uwot (https://github.com/jlmelville/uwot). Features were scaled to unit variance and the 15 nearest neighbors were considered for embedding. UMAP coordinates were then exported for visualization using ggplot2 (76).

FlowSOM (35) uses self-organizing maps to efficiently categorize cytometry data into nonoverlapping cell populations. Clustering was performed with a target cluster number of 100 and metaclustering with a target number of 45. This approach purposefully overclustered the data to resolve potentially small subsets, a trade-off that can split phenotypically similar cells into more than 1 population (77). Overclustering was addressed by manual inspection and merging of phenotypically similar populations; in this way, each T cell was classified into 1 of 34 unique clusters. Names were assigned manually using activation, lineage, and memory markers to broadly categorize each T cell cluster; when more than 1 cluster was placed into the same category, clusters were given an accessory label to highlight their unique phenotype or property (e.g., skin-homing, indicated by the expression of CLA). The ComplexHeatmap package (78) was used to visualize T cell cluster phenotypes; the arcsine-transformed signal intensity of each marker was independently scaled using a 0–1 transformation across all 34 clusters.

### CyTOF data analysis

For differential abundance analysis of T cell clusters, we followed the workflow laid out in Nowicka et al. (73). FlowSOM cluster cell counts were modeled linearly with time point as a dependent categorical variable and volunteer as a fixed effect using the diffcyt (37) implementation of edgeR (36). The edgeR functions automatically normalize cluster counts for the total number of cells and improve statistical power by sharing information on cluster count variance between clusters. Using moderated *F* tests, pairwise comparisons were performed relative to baseline and clusters with an FDR of less than 0.05 and absolute fold-change greater than 2 were deemed to vary significantly through time. We also assessed whether marker expression varied significantly through time. To do this, we merged clusters of the same lineage according to their expression of CD4, CD8α, Vδ2, and Vα7.2. CD4^+^ and CD8^+^ T cells were then split into naive, effector, effector memory, central memory, and Temra subsets based on their expression of the markers CD45RA, CD45RO, CD57, and CCR7. All Tregs (CD25^hi^CD127^–^) were merged into a single cluster as were all double-negative, γδ T, and MAIT cells. Linear regression models were fit using limma (which is optimized for continuous data) incorporating time point, volunteer, and acquisition batch as covariates. To independently assess differential marker expression (relative to baseline), moderated *t* tests were used to test regression coefficients; a shift in median expression of at least 10% and an FDR of less than 0.05 were required for significance. Results were visualised using ComplexHeatmap with row-wise *z* score–transformed marker intensities shown for each subset.

### Comparison to *P. falciparum*

To compare the immune response induced by *P. vivax* versus *P. falciparum*, we used data from 2 previously conducted CHMI trials (VAC063 and VAC063C); the VAC063 trial is described in full in Minassian et al. (42) and the VAC063C trial is reported in Salkeld et al. (43). In brief, 13 malaria-naive adult volunteers (10 in VAC063 and 3 in VAC063C) were inoculated with *P. falciparum* (clone 3D7) by intravenous injection of infected red cells (452–857 parasites per volunteer). The inoculum derived from a cryopreserved stabilate that was thawed, washed, and diluted under aseptic conditions immediately before challenge. Starting 1 day after infection, volunteers attended clinic for assessment and blood sampling every 12 hours, and circulating parasite density was measured in real time by qPCR (target gene = 18S ribosomal RNA). At diagnosis (symptomatic with more than 5,000 parasites/mL or asymptomatic above 10,000 parasites/mL) volunteers were treated with either Riamet or Malarone (if Riamet was contraindicated). Blood for immunological analyses was collected in EDTA tubes by venipuncture at baseline, diagnosis, T6, and C90 (memory time point). To analyze T cell activation, whole blood samples were preserved during VAC063C and processed for CyTOF exactly as described for *P. vivax*. Data processing was also performed analogously to VAC069A and FlowSOM was used to place each T cell into a phenotypically unique cluster. Note that clustering was performed independently on the VAC069A and VAC063C data sets. For each volunteer, the proportion of activated CD4^+^ T cells and Tregs was then calculated by summing the frequency of all clusters with a CD38^hi^Bcl2^lo^ phenotype. To compare parasite species–specific differences in T cell activation, we used a 2-tailed Wilcoxon’s rank-sum exact test.

### Statistics

Analysis was carried out in R (v3.6.3; https://cran.rstudio.com/) unless otherwise stated.

#### Multiplexed plasma analysis.

Protein concentrations were log_10_-transformed and linear regression models were fit separately for each analyte. For the *P. vivax* data set, linear regression models were fit including time point and volunteer identity as fixed effects using the R package stats. To compare *P. vivax*– and *P. falciparum*–infected volunteers, mixed-effects models were fit, including time point and parasite species as fixed effects and volunteer identity as a random effect using lme4. Linear hypothesis testing was then performed using multcomp. For analysis of *P. vivax*, *t* tests were used to test the significance of model coefficients (each time point versus baseline). Similarly, to compare *P. vivax* versus *P. falciparum*, we used *z* tests. *P* values were adjusted for multiple testing using Benjamini-Hochberg correction and an FDR of less than 0.05 was considered significant.

#### Whole blood RNA sequencing.

DESeq2 (29) was used to identify DEGs at each time point (cf baseline) and adjusted *P* values were obtained using Wald’s test (incorporating time point and volunteer identity as covariates). Analysis was performed independently on the *P. vivax* and *P. falciparum* data sets. In both cases, DEGs were classified as those with an absolute fold-change greater than 1.5 and an FDR of less than 0.05. Genes with multiple differentially expressed transcripts were filtered to retain the transcript with the lowest adjusted *P* value. GO analysis was carried out in Cytoscape (version 3.8.0) using the ClueGO plugin (version 2.5.7) (30, 31) and networks were constructed by combining the lists of DEGs from volunteers infected with *P. vivax* and *P. falciparum*. Importantly, for every GO term, information on what fraction of associated genes was derived from each list was retained. Any GO term containing more than 65% associated genes from a single volunteer cohort was considered to be enriched in that infection model; otherwise, GO terms were considered to be shared.

#### Pearson’s correlation.

The fold-change of each T cell cluster and plasma analyte was calculated using raw cluster percentages or plasma concentrations, respectively. For each feature, these were calculated at diagnosis or T6 (relative to baseline) according to their largest absolute fold-change. All data were log_2_-transformed and Pearson’s correlation was performed using the cor function from the stats package. Correlation coefficients were then used for hierarchical clustering by Euclidean distance using ComplexHeatmap.

### Study approval

The CHMI trials were sponsored by the University of Oxford and received ethical approval from the UK NHS Research Ethics Service — VAC069A (South Central Hampshire A, reference 18/SC/0577), VAC063 (Oxfordshire Research Ethics Committee A, reference 16/SC/0345), and VAC063C (South Central Oxford A, reference 18/SC/0521). All trials were registered on ClinicalTrials.gov (NCT03797989, NCT02927145, and NCT03906474, respectively) and were conducted in the UK at the Centre for Vaccinology and Tropical Medicine (University of Oxford). Trials were conducted according to the principles of the current revision of the Declaration of Helsinki (2008) and in full conformity with the ICH Guidelines for Good Clinical Practice. Volunteers signed written consent forms and consent was checked prior to each CHMI.

### Data availability

RNA sequencing data from VAC069A have been deposited in the European Genome-phenome Archive (EGA) and are accessible through accession number EGAS00001003847. Sequencing data from VAC063/VAC063C have been deposited in NCBI’s Gene Expression Omnibus and are accessible through accession number GSE172450. CyTOF data are available at http://flowrepository.org/ and can be accessed through experiment numbers FR-FCM-Z3HA (VAC069A) and FR-FCM-Z47Z (VAC063C). Raw clinical data (including parasite growth curves, full blood counts, and blood chemistry) are available in the Supporting Data Values file.

## Author contributions

FAB and PJS designed the research study. FAB, DMS, MM, ACH, and NJE conducted experiments. FAB, DMS, MM, YT, TAR, ACH, and NJE acquired data. FAB, DMS, ACH, AI, GN, and PJS analyzed the data. YT, TAR, SES, JRB, AMM, and SJD provided reagents. AK, JCR, AMM, SJD, and PJS provided project management and oversight. FAB and PJS wrote the manuscript; all authors edited and approved the final manuscript.

## Supplementary Material

Supplemental data

ICMJE disclosure forms

Supplemental table 1

Supplemental table 2

Supporting data values

## Version 1 08/24/2023

In-Press Preview

## Version 2 10/16/2023

Electronic publication
