# Supplementary material for: A systematic analysis of the human immune response to Plasmodium vivax
Source: J Clin Invest. 2023 Oct 16;133(20):e152463. doi: 10.1172/JCI152463 (PMC10575735; doi:10.1172/JCI152463)
Supplement: Supplemental data [file jci-133-152463-s091.pdf]

Supplemental material for a systematic analysis of the human immune response to *Plasmodium vivax*

Florian A. Bach,[1] Diana Muñoz Sandoval,[1,2] Michalina Mazurczyk,[3] Yrene Themistocleous,[4] Thomas A. Rawlinson,[4] Adam C. Harding,[1] Alison Kemp,[5] Sarah E. Silk,[4,6] Jordan R. Barrett,[4,6] Nick J. Edwards,[4] Alasdair Ivens,[1] Julian C. Rayner,[5] Angela M. Minassian,[4,6] Giorgio Napolitani,[3] Simon J. Draper,[4,6] Philip J. Spence[1]

[1]Institute of Immunology and Infection Research, University of Edinburgh, UK. [2]Instituto de Microbiologia, Universidad San Francisco de Quito, Ecuador. [3]MRC Human Immunology Unit, Weatherall Institute of Molecular Medicine, University of Oxford, UK. [4]The Jenner Institute, University of Oxford, UK. [5]Cambridge Institute for Medical Research, University of Cambridge, UK. [6]Department of Biochemistry, University of Oxford, UK.

## Supplemental methods

### Multiplexed plasma analysis

Whole blood was centrifuged at 1000g for 5 minutes to separate cellular components and plasma. Plasma was then aspirated and centrifuged at 2000g for 10 minutes to remove platelets. Avoiding the pellet, supernatants were aliquoted and snap-frozen on dry ice before storage at -80°C until further processing. Plasma samples from baseline, diagnosis, T6 and memory phase were thawed on ice before centrifuging at 1000g for 1 minute to remove potential protein aggregates. The concentration of 39 different analytes was then measured by running every sample across four different custom Legendplex assays from Biolegend, according to the manufacturer's instructions. Filter plates with samples and concentration standards were then acquired on a LSRFortessa flow cytometer (BD). FCS files were processed using Legendplex software (v7.1), which automatically interpolates a standard curve using the plate-specific standards and calculates analyte concentrations for each sample. Samples from v09 were excluded after failing QC and statistical analysis of the remaining 5 volunteers was carried out in R (v3.6.3). Using the stats package we fit linear regression models using restricted maximum likelihood for each analyte with log10 transformed analyte concentrations as response variable and time-point and volunteer as categorical fixed effects. Linear hypothesis testing via pairwise comparisons (each time-point versus baseline) was then performed using the glht function from the multcomp package and adjusted for multiple testing (Benjamini & Hochberg). An FDR $< 0.05$ was considered significant. Results were visualised using ComplexHeatmap, inspired by the plotDiffHeatmap function of CATALYST (1) and ggplot2.

**Whole blood RNA-sequencing**

Whole blood was added to Tempus reagent (Applied Biosystems) at a ratio of 1:2 within 60-minutes of blood draw and stored at -80°C. RNA extraction was performed using the Tempus Spin RNA isolation reagent kit (Applied Biosystems) according to the manufacturer's instructions. Briefly, lysed blood samples were thawed and centrifuged at 3000g for 30 minutes at 4°C to pellet nucleic acids. Pellets were resuspended in RNA purification resuspension solution and centrifuged on a silica column to remove non-nucleic acid contaminants. After washing, the column was incubated for two minutes at 70°C before eluting nucleic acids. The eluate was then subjected to DNA digestion using the RNA Clean and Concentrator-5 kit (Zymo Research). Purified RNA was eluted in 30μl DNAse/RNAse-free water; quantification and quality control were carried out on a Qubit (Thermo Fisher Scientific) and Bioanalyzer (Agilent Technologies), respectively. All samples were diluted to a concentration of approximately 20-40ng $\mu l^{-1}$ and shipped to the Wellcome Sanger Institute for library preparation and sequencing. Libraries were constructed using the NEBNext Ultra II RNA library prep kit on an Agilent Bravo WS automation system followed by 14 cycles of PCR using KAPA HiFi HotStart DNA polymerase. Libraries were then pooled in equimolar amounts and 75bp paired end (PE) reads were generated on the Illumina HiSeq v4 according to the manufacturer's standard protocol (~ 35 million PE reads per sample).

FASTQ files were quality assessed using FASTQC, reads were aligned to the human transcriptome (Ensembl, release 98) using bowtie2 (v2.2.7) (2) and after alignment globin reads were discarded. DESeq2 was used for differential gene expression analysis; differentially expressed genes were classified as those with an adj $p < 0.05$ and an absolute fold-change > 1.5. Genes with multiple differentially expressed transcripts were filtered to retain the transcript with the lowest adj p value. Gene ontology analysis was performed in Cytoscape (version 3.8.0) using the ClueGO plugin (version 2.5.7). The ontologies GO Biological Process-EBI-uniprot-GOA and GO Molecular Function-EBI-uniprot-GOA were used (updated 08.05.2020). ClueGO networks were constructed using GO term levels 5-11, GO fusion = true and a lower cut-off of 3 genes (or 5% associated genes). The lower bound for connecting GO terms with shared genes was set at a kappa score of 0.4.

**Stimulation of whole blood with PMA/Ionomycin**

Whole blood (500μl) was diluted 1 in 5 with RPMI plus 2mM L-Glutamine and cultured for 6-hours at 37°C / 5% $CO_2$ in 6-well ultra-low attachment plates (Corning). Stimulated samples received 50ng $ml^{-1}$ PMA and 1μg $ml^{-1}$ Ionomycin; after 2-hours of culture 5μg $ml^{-1}$ Brefeldin A was added to both stimulated and unstimulated samples. At the end of the culture period cell suspensions were transferred to a 15ml polypropylene tube, centrifuged at 400g for 5 minutes (room temperature) and resuspended in 400μl RPMI containing 2mM L-Glutamine and 5mM EDTA. Samples were then stabilised by adding 400μl whole blood preservation buffer (Cytodelics AB)

and inverted to mix. After incubating for 10 minutes at room temperature each sample was split equally into two cryovials and snap frozen on dry ice. Samples were stored long-term at -80°C.

**Analysis of cytokine production by flow cytometry**

Cryopreserved whole blood samples were thawed in a water bath at 37°C and then fixed and red cell lysed using the whole blood preservation kit (Cytodelics AB). After lysis, cells were washed with flow buffer (PBS containing 2% FBS and 5mM EDTA), centrifuged at 800g for 5 minutes and resuspended in 450μl PBS supplemented with 125U $ml^{-1}$ sodium heparin. Samples were incubated for 20 minutes, washed and centrifuged (as before) and resuspended in 100μl flow buffer containing 3μl human TruStain FcX (BioLegend). After a brief 5 minute incubation fluorophore-conjugated antibodies against CD3 (OKT3) and CD38 (HIT2) were added and cells were incubated in the dark for 20 minutes. Samples were then washed (once with flow buffer and once with PBS), incubated for 30 minutes in 200μl intracellular fixation buffer (eBioscience) and washed two times with permeabilisation buffer (eBioscience). Fixed and permeabilised cells were incubated with sodium heparin and TruStain FcX as before (keeping cells in permeabilisation buffer) and then resuspended in 100μl permeabilisation buffer containing fluorophore-conjugated antibodies against intracellular targets (Granzyme B (GB11), IFNγ (B27), IL-2 (MQ1-17H12), IL-4 (MP4-25D2), IL-10 (JES3-9D7), IL-17A (BL168), IL-21 (3A3-N2), IL-22 (2G12A41) and TNF (Mab11)). Staining was carried out in the dark for 45 minutes after which cells were washed two times with permeabilisation buffer and resuspended in flow buffer for acquisition on a LSRFortessa running FACSDiva software (v8). Note that all incubation and wash steps were carried out at room temperature, all antibodies were purchased from BioLegend and samples were passed through a 40μm filter prior to acquisition. Downstream data analysis was performed in R (v4.1.3) with a compensation matrix generated using the flowCore package (3). Event QC was performed with the flow_auto_qc function of FlowAI (4) to automatically exclude outliers (due to flow-rate and dynamic range irregularities) and gating was performed in FlowWorkspace (5) using the flowClust.2d algorithm. Plotting of summary data was carried out in ggplot2.

**Comparison to *P. falciparum***

*Multiplexed plasma analysis*: to measure the acute phase response to *P. falciparum* platelet-depleted plasma was prepared during VAC063/VAC063C exactly as described for VAC069A. We then ran the *P. falciparum* and *P. vivax* CHMI samples together across four custom Legendplex assays to measure 39 biomarkers of inflammation, coagulation and oxidative stress. Samples were randomised and analysed contemporaneously to remove possible batch effects between the two infection models. Samples from v1040 (*P. falciparum*) and v09 (*P. vivax*) were excluded after failing QC and statistical analysis of the remaining volunteers was

carried out by linear regression on log10 transformed concentrations. For each analyte a linear mixed effects model was fit using the lmer function from the lme4 package (v1.1-27.1), including volunteer identity as a random effect, with the formula concentration~timepoint+species+(1|volunteer). Linear hypothesis testing was performed with the glht function from the multcomp package (v1.4-17) to compare parasite species specific differences in analyte concentration at diagnosis and T6 (relative to baseline); p values were calculated using z-tests and adjusted for multiple comparisons (Benjamini & Hochberg). Adjusted p values $< 0.05$ were considered significant. Note that samples were not collected at T6 during VAC063 and therefore n = 3 for *P. falciparum* at this time-point.

*Whole blood RNA-sequencing*: whole blood samples from VAC063/VAC063C were processed exactly as described for *P. vivax* CHMI. Libraries were then prepared using the TruSeq stranded mRNA library prep kit (Illumina) and sequenced on the NovaSeq 6,000 Illumina platform to yield 50 bp PE reads. Data processing and analysis was carried out analogously to VAC069A to identify differentially expressed genes at diagnosis and T6 (versus baseline). At each time-point we combined the lists of differentially expressed genes from volunteers infected with *P. vivax* and *P. falciparum* to construct ClueGO networks. We then asked whether GO term enrichment was predominantly derived from one dataset to examine parasite species-specific differences in the host response to infection. To define a threshold for meaningful enrichment we relied on Chebyshev's inequality. This rule states that, for a wide range of probability distributions, at least $1 - 1/k^2$ of the distribution's values are less than $k$ standard deviations away from the mean. For example, 95% of values must be less than or equal to ~ 4.5 standard deviations from the mean. Applying this heuristic to our GO analysis showed that the middle 95% of the distribution of GO terms were enriched by less than or equal to 15% from either dataset. As such, we considered any GO term containing > 65% associated genes from a single volunteer cohort to be in the top 5% of GO terms predominantly derived from one dataset. In all other cases, GO terms were considered to be shared.

Supplemental references

1. Crowell H, et al. CATALYST: Cytometry dATa anALYSis Tools. *R/Bioconductor.* 2020;version 1.14.0.
2. Langmead B, and Salzberg SL. Fast gapped-read alignment with Bowtie 2. *Nat Methods.* 2012;9(4):357-9.
3. Hahne F, et al. flowCore: a Bioconductor package for high throughput flow cytometry. *BMC Bioinformatics.* 2009;10:106.
4. Monaco G, et al. flowAI: automatic and interactive anomaly discerning tools for flow cytometry data. *Bioinformatics.* 2016;32(16):2473-80.
5. Finak G, et al. QUAliFiER: an automated pipeline for quality assessment of gated flow cytometry data. *BMC Bioinformatics.* 2012;13:252.

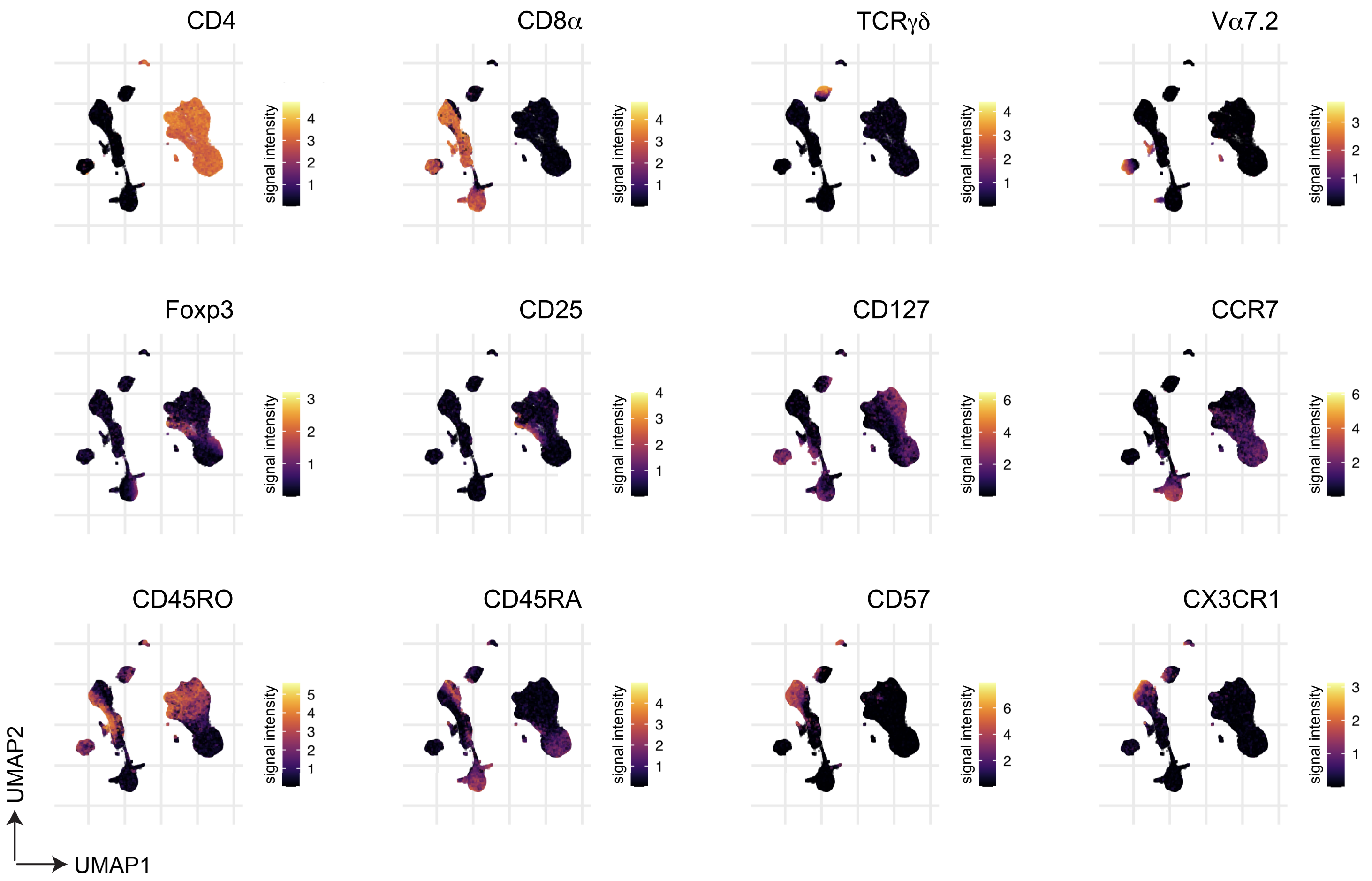


**Supplemental Figure 1.** Expression of lineage and memory markers across the UMAP projection; used to pin-point the location of each major T cell subset. Data from all volunteers were concatenated (n = 6) and split by time-point. The arcsine transformed signal intensity is plotted for each marker and is shown at T6.

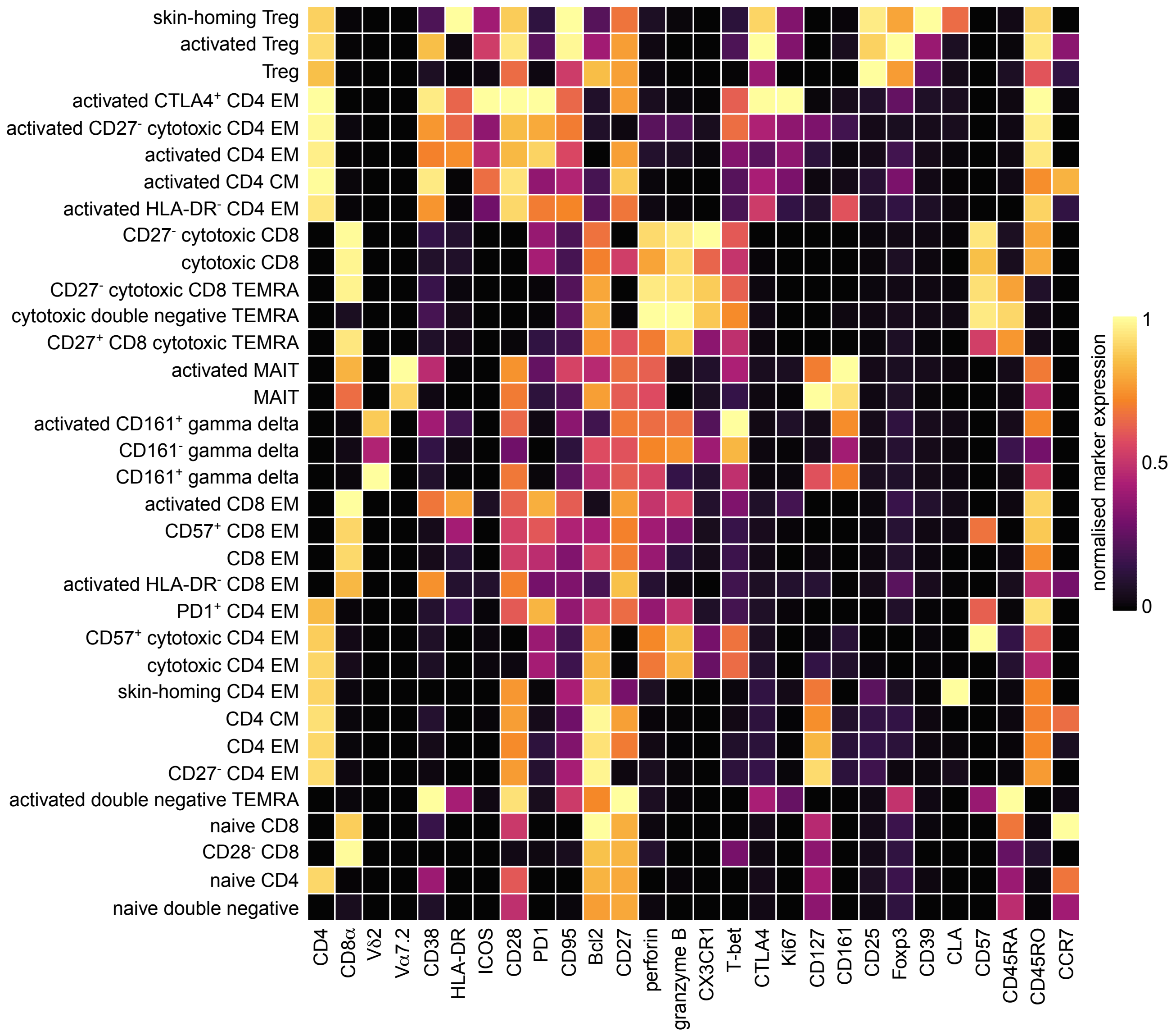


**Supplemental Figure 2.** Phenotype of each unique T cell cluster. Heatmap showing normalised median expression values of all markers used for clustering in each of the 34 T cell clusters. Marker expression was normalised according to expression intensity across all cells; a value of 1 indicates the highest level of expression across the entire dataset and a value of 0 the lowest. Names were assigned manually using activation, lineage and memory markers to broadly categorise each T cell cluster; when more than one cluster was placed into the same category (e.g. activated CD4 EM) clusters were given an accessory label to highlight their unique phenotype or property (e.g. skin-homing, indicated by the expression of CLA). Note that data from all volunteers and time-points were concatenated for clustering.

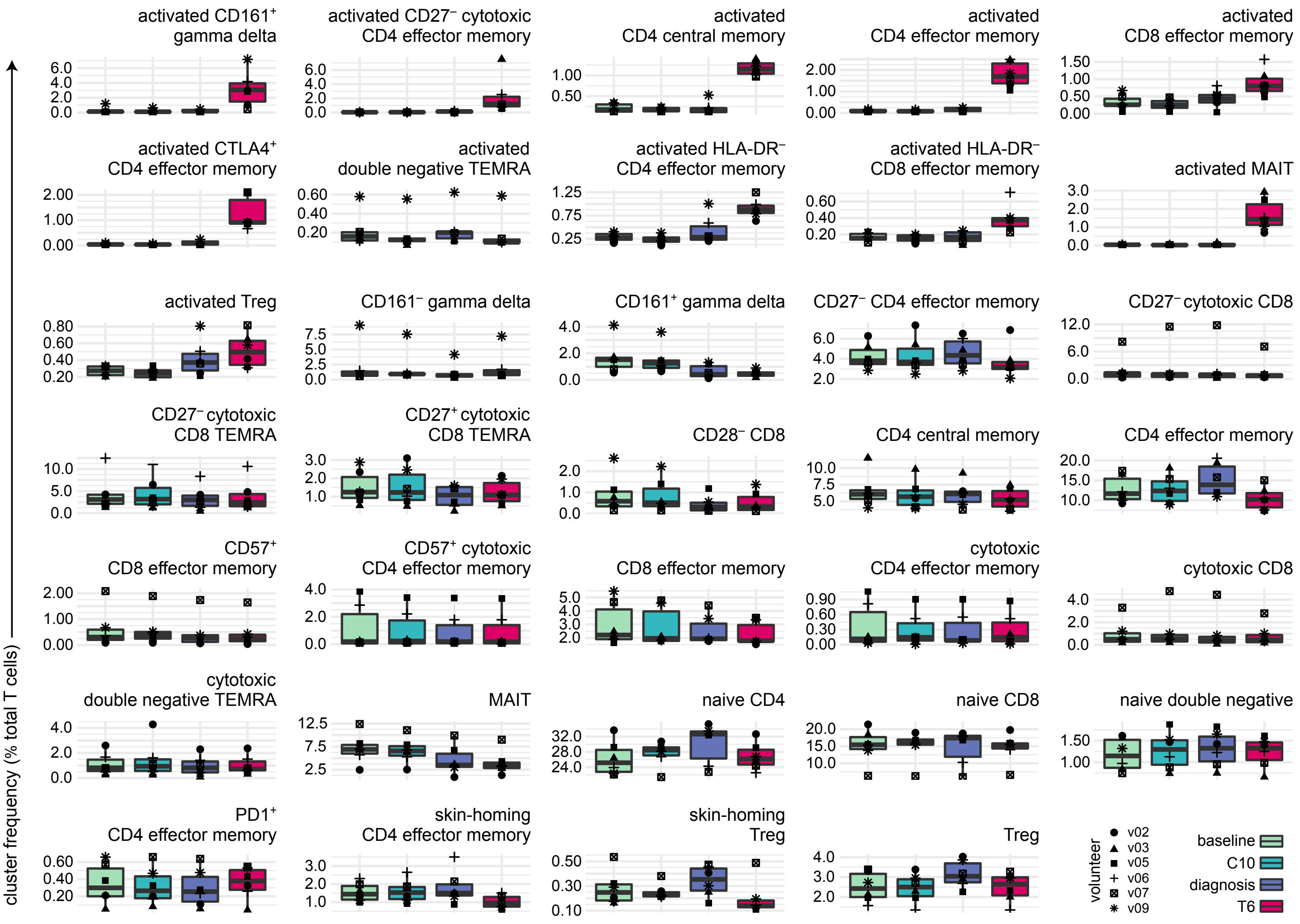


**Supplemental Figure 3.** Frequency of each T cell cluster during and after infection. Every unique T cell cluster is shown as the proportion of total T cells at each time-point, and each symbol represents one volunteer (n = 6). These data underpin the heatmap showing differentially abundant clusters in Figure 4.

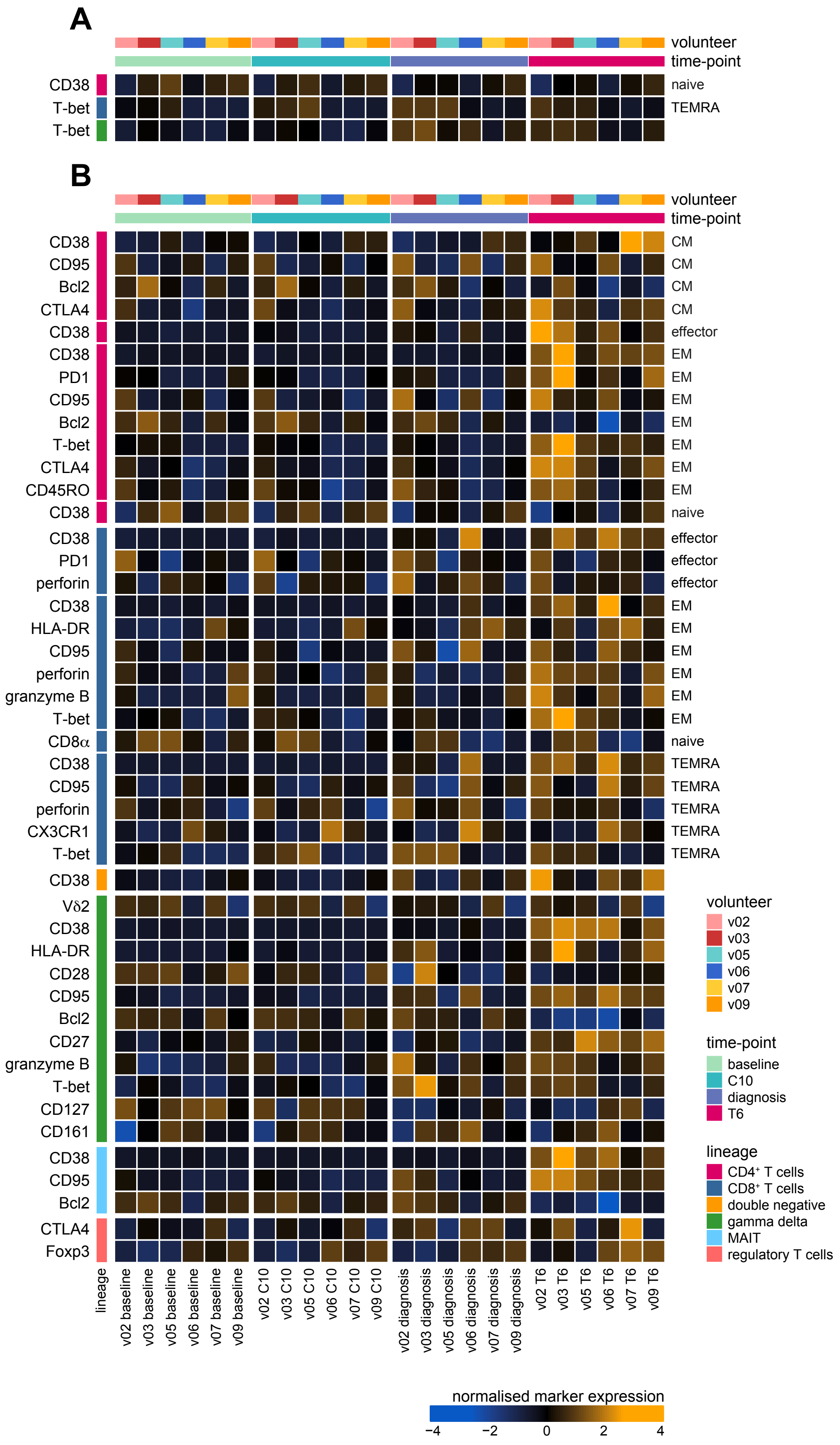


**Supplemental Figure 4.** Differential marker expression on T cells during and after infection. (**A-B**) We assessed whether marker expression significantly varies through time in all major T cell subsets. First, T cell clusters belonging to the same lineage were merged and then $CD4^+$ and $CD8^+$ T cells were split into naive, effector, effector memory (EM), central memory (CM) and TEMRA subsets. Next, linear models were used to independently assess differential marker expression in each subset at each time-point (relative to baseline); a shift in median expression of at least 10% and an FDR < 0.05 were required for significance. Shown are all subset/marker pairs that were called as significant at diagnosis (A) and T6 (B). Data are presented as row-wise z-score marker intensities and each column represents a sample (n = 6). Colour codes to the left of the heatmap indicate lineage and subset names are listed for $CD4^+$ and $CD8^+$ T cells to the right of the heatmap.

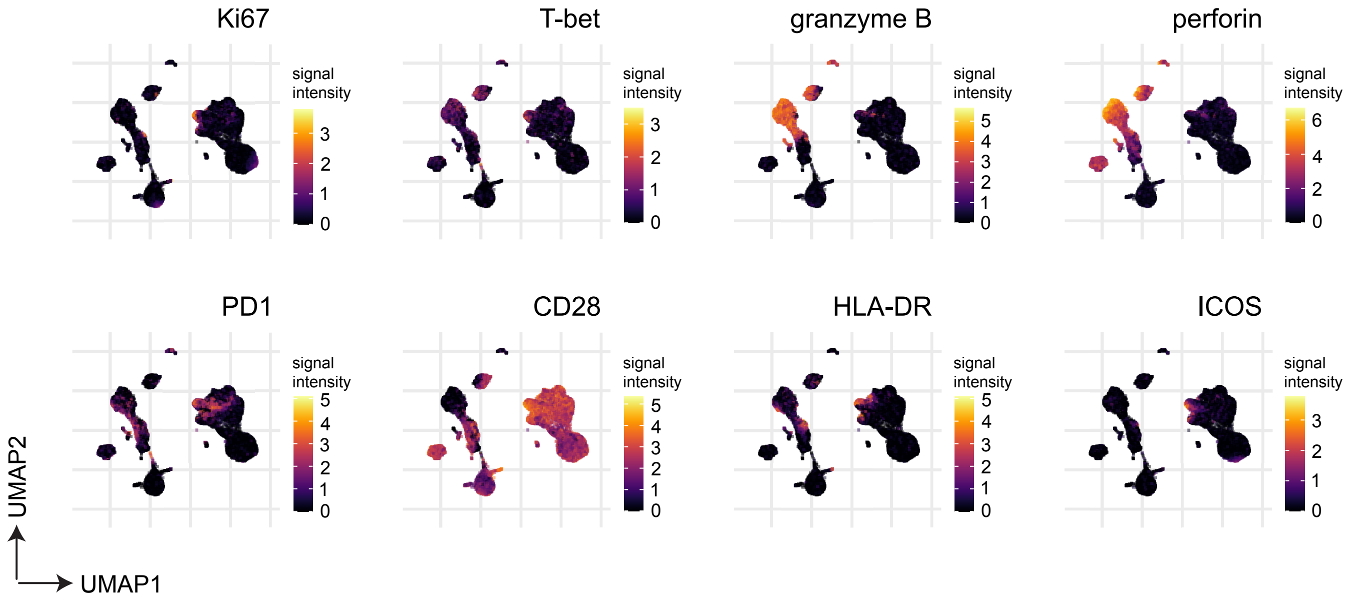


**Supplemental Figure 5.** Expression of activation, proliferation and differentiation markers across the UMAP projection at T6. Data from all volunteers were concatenated (n = 6) and split by time-point; the arcsine transformed signal intensity is plotted for each marker.

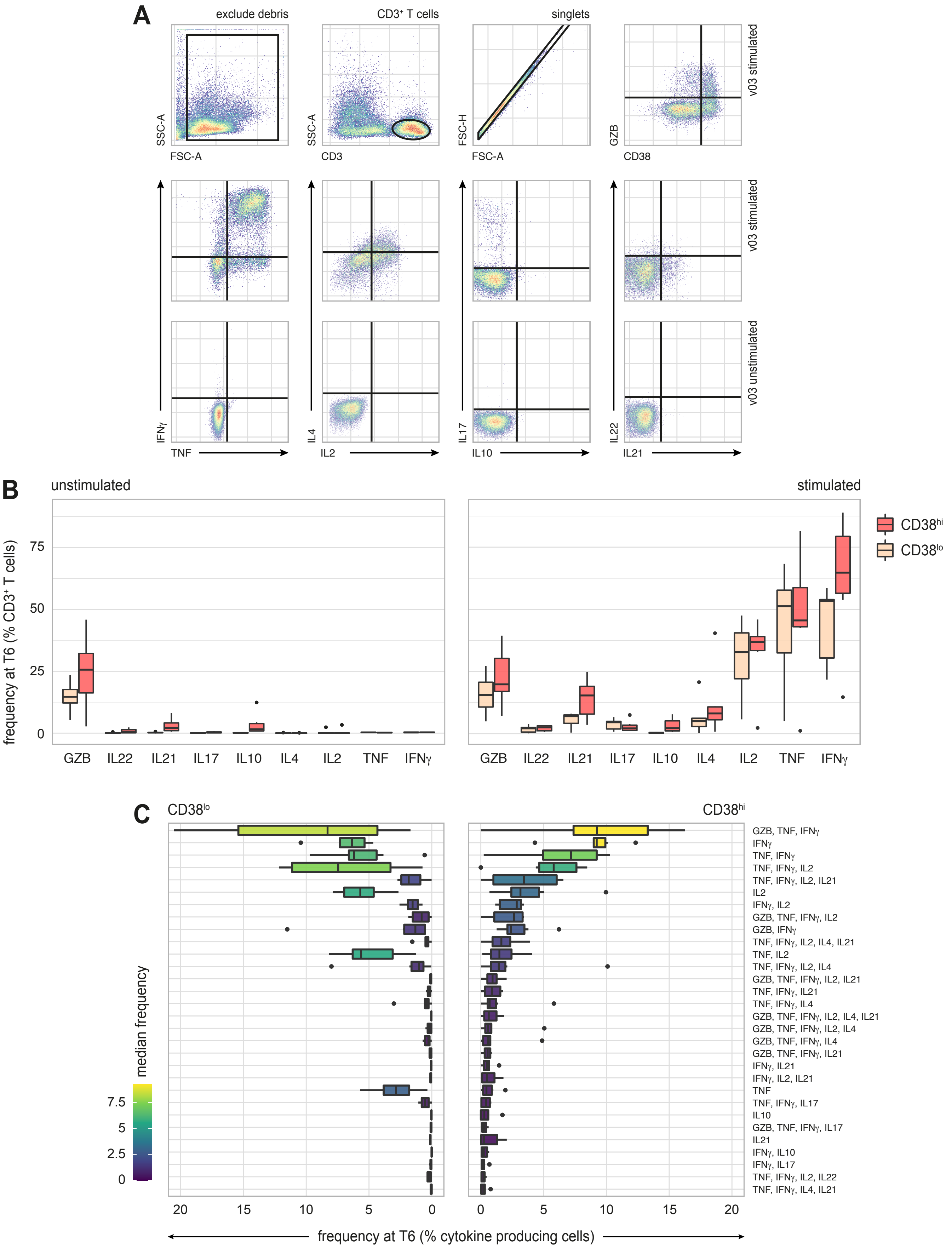


**Supplemental Figure 6.** Cytokine production by activated (CD38hi) and resting (CD38lo) T cells following mitogenic stimulation. Whole blood was cultured with PMA/Ionomycin immediately *ex vivo* (without cryopreservation) at T6 and cytokine production quantified by flow cytometry. (**A**) CD3⁺ singlets were split into CD38hi and CD38lo subsets by manual gating and the expression of Granzyme B and each of 8 cytokines (covering $T_H1$, $T_H2$, $T_H17$ and $T_{FH}$ differentiation) were assessed. Data from a representative volunteer (v03) are shown. (**B**) The percentage of CD38hi and CD38lo CD3⁺ T cells that are Granzyme B or cytokine positive at T6 is shown for unstimulated (control) cultures and those stimulated with PMA/Ionomycin. (**C**) Boolean gating was then used to examine the co-expression of Granzyme B and each of the 8 cytokines in stimulated CD38hi and CD38lo T cells. The top 20 subsets for CD38hi cells are shown. In (B-C) box (median and IQR) and whisker (1.5x upper or lower IQR) plots are shown with outliers as dots (n = 6). There were no significant differences between CD38hi and CD38lo T cells for any marker (or combination of markers) assessed (adj $p > 0.05$ by Wilcoxon signed-rank test (two-tailed) with Benjamini-Hochberg). Note that PMA/Ionomycin stimulation does not upregulate CD38 expression *in vitro* and therefore CD38hi T cells were activated by *P. vivax in vivo*.

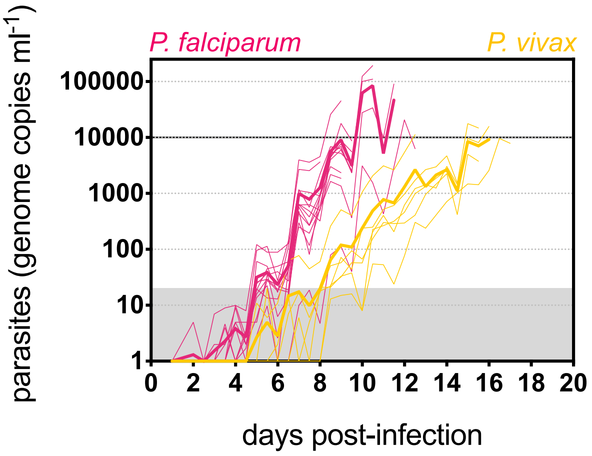


**Supplemental Figure 7.** Infection dynamics during CHMI. Circulating parasite density was determined twice daily by qPCR during the VAC063/VAC063C trials (*P. falciparum*) and the VAC069A study (*P. vivax*). Each line represents a single volunteer (bold lines show the mean) and the limit of quantification (20 genome copies ml$^{-1}$) is denoted by the shaded area (n = 13 for *P. falciparum* and n = 6 for *P. vivax*).
