# Supplementary material for: A systematic analysis of the human immune response to Plasmodium vivax
Source: J Clin Invest. 2023 Oct 16;133(20):e152463. doi: 10.1172/JCI152463 (PMC10575735; doi:10.1172/JCI152463)
Supplement: Supplemental table 2 [file jci-133-152463-s094.pdf]

isotype range	marker	location	antibody clone	source
89Y	CD45	cell surface	HI30	Fluidigm (3089003B)
111-116 Cd	CD14	cell surface	TuK4	Thermo (Q10056)
115 In	CD57	cell surface	HCD57	Biolegend (322325)
141 Pr	HLA-DR	cell surface	L243	Biolegend (307602)
142 Nd	Bcl2	nuclear	100	Biolegend (658702)
143 Nd	CD45RA	cell surface	HI100	Biolegend (304102)
144 Nd	granzyme B	intracellular	GB11	Abcam (ab103159)
145 Nd	CD4	cell surface	RPA-T4	Biolegend (300502)
146 Nd	Vδ2	cell surface	123R3	Miltenyi (130-095-795)
147 Sm	CD20	cell surface	2H7	Biolegend (302302)
148 Nd	ICOS	cell surface	DX29	BD biosciences (557801)
149 Sm	CXCR5	cell surface	RF8B2	BD biosciences (552032)
150 Nd	CD95	cell surface	DX2	Biolegend (305602)
151 Eu	CD103	cell surface	Ber-ACT8	Fluidigm (3151011B)
152 Sm	TCRγδ	cell surface	11F2	Fluidigm (3152008B)
153 Eu	Vα7.2	cell surface	3C10	Fluidigm (3153024B)
154 Sm	Tim-3	cell surface	F38-2E2	Fluidigm (3154010B)
155 Gd	PD1	cell surface	EH12.2H7	Fluidigm (3155009B)
156 Gd	CD161	cell surface	HP-3G10	Biolegend (339902)
158 Gd	CD27	cell surface	L128	Fluidigm (3158010B)
159 Tb	Foxp3	nuclear	259D/C7	Fluidigm (3159028A)

160 Gd	CTLA4	intracellular	14D3	eBioscience (14-1529-82)
161 Dy	T-bet	nuclear	4B10	Fluidigm (3161014B)
162 Dy	integrin β7	cell surface	FIB504	Fluidigm (3162026B)
163 Dy	CD28	cell surface	L293	BD biosciences (348040)
164 Dy	Ki67	nuclear	Ki-67	Biolegend (350502)
165 Ho	CD45RO	cell surface	UCHL1	Biolegend (304202)
166 Er	CD56	cell surface	HCD56	Biolegend (318302)
167 Er	CCR7	cell surface	150503	R&D systems (MAB197-100)
168 Er	CD127	cell surface	A019D5	Biolegend (351302)
169 Tm	CD38	cell surface	HIT2	Biolegend (303502)
170 Er	CD3	cell surface	UCHT1	Biolegend (300402)
171 Yb	CD49d	cell surface	9F10	Biolegend (304302)
172 Yb	CD25	cell surface	BC96	Biolegend (302602)
173 Yb	CD39	cell surface	A1	Biolegend (328202)
174 Yb	CLA	cell surface	HECA-452	Biolegend (321302)
175 Lu	perforin	intracellular	B-D48	Fluidigm (3175004B)
176 Yb	CX3CR1	cell surface	2A9-1	Biolegend (341602)
191/193 Ir	DNA intercalator	na	na	Fluidigm (201192A)
198 Pt	CD8α	cell surface	RPA-T8	Biolegend (301002)
209Bi	CD16	cell surface	3G8	Fluidigm (3209002B)

Supplemental Table 2. Antibody panel for T cell function and fate in vivax malaria; includes information on the antibody clone and heavy metal conjugate.
